# Supplementary material for: Reference genome and transcriptome informed by the sex chromosome complement of the sample increase ability to detect sex differences in gene expression from RNA-Seq data
Source: Biol Sex Differ. 2020 Jul 21;11:42. doi: 10.1186/s13293-020-00312-9 (PMC7374973; doi:10.1186/s13293-020-00312-9)
Supplement: Supplementary file 2 — Additional file 2: Histogram of sample reported age. For each tissue, whole blood, brain cortex, breast, liver, and thyroid, male XY and female XX samples were age matched perfectly between age 55 to 70. Females are shown in blue and males are shown in lime green. Since the samples were aged perfectly the histogram bars show only the overlap of female and male samples is a mix color of the blue and lime green. [file 13293_2020_312_MOESM2_ESM.pdf]

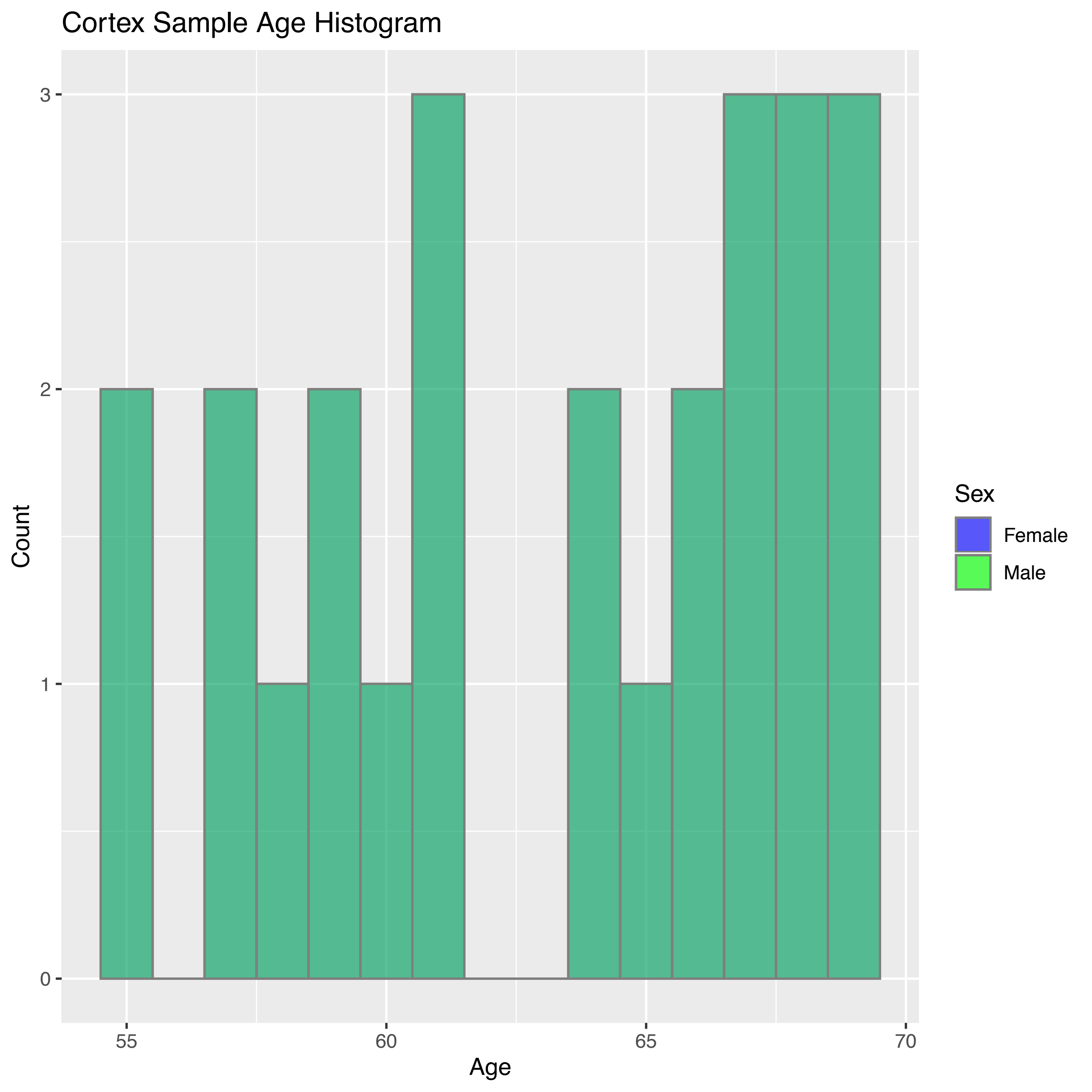

Cortex Sample Age Histogram
Count
Age
0
1
2
3
55
60
65
70
Sex
Female
Male

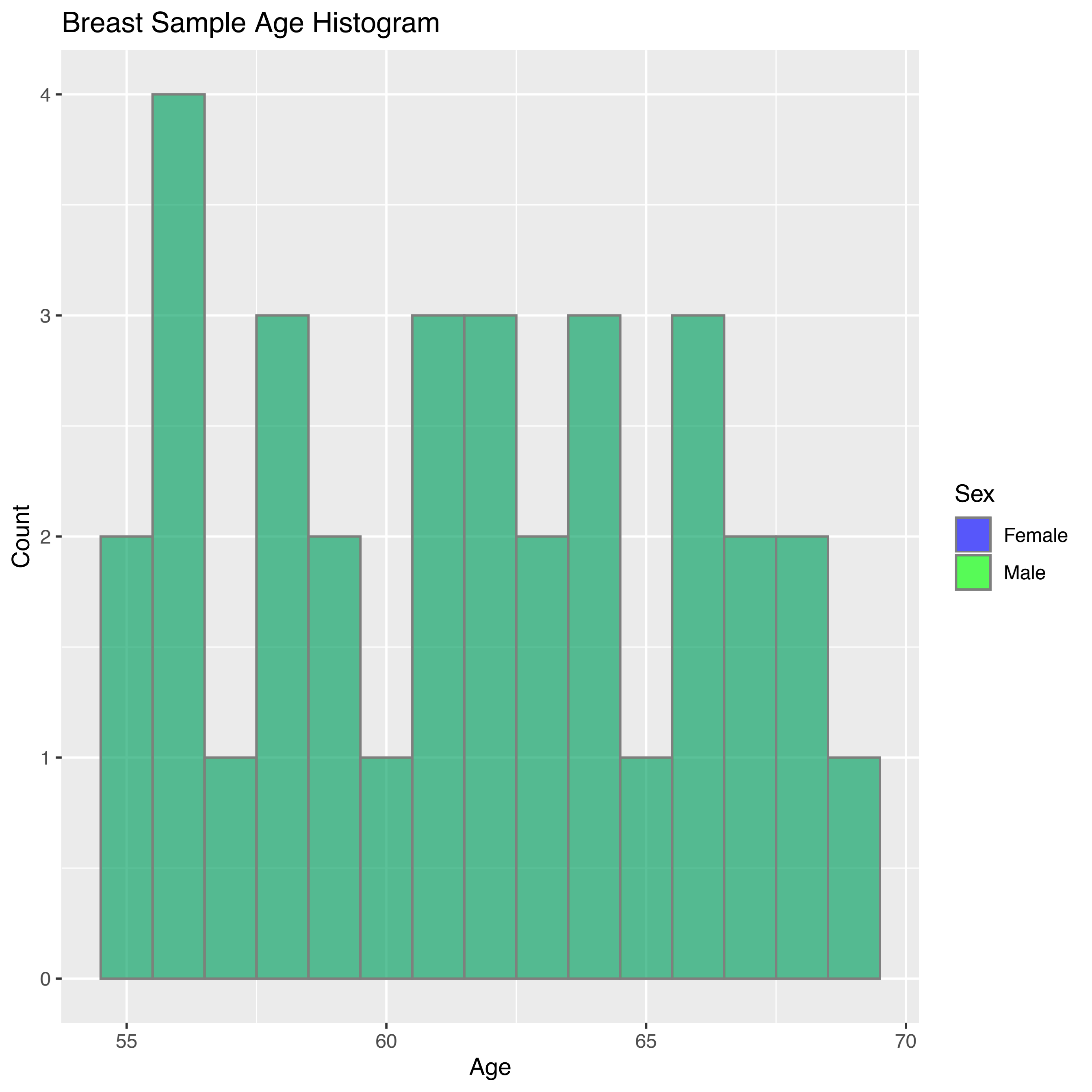

Breast Sample Age Histogram
Count
Age
0
1
2
3
4
55
60
65
70
Sex
Female
Male

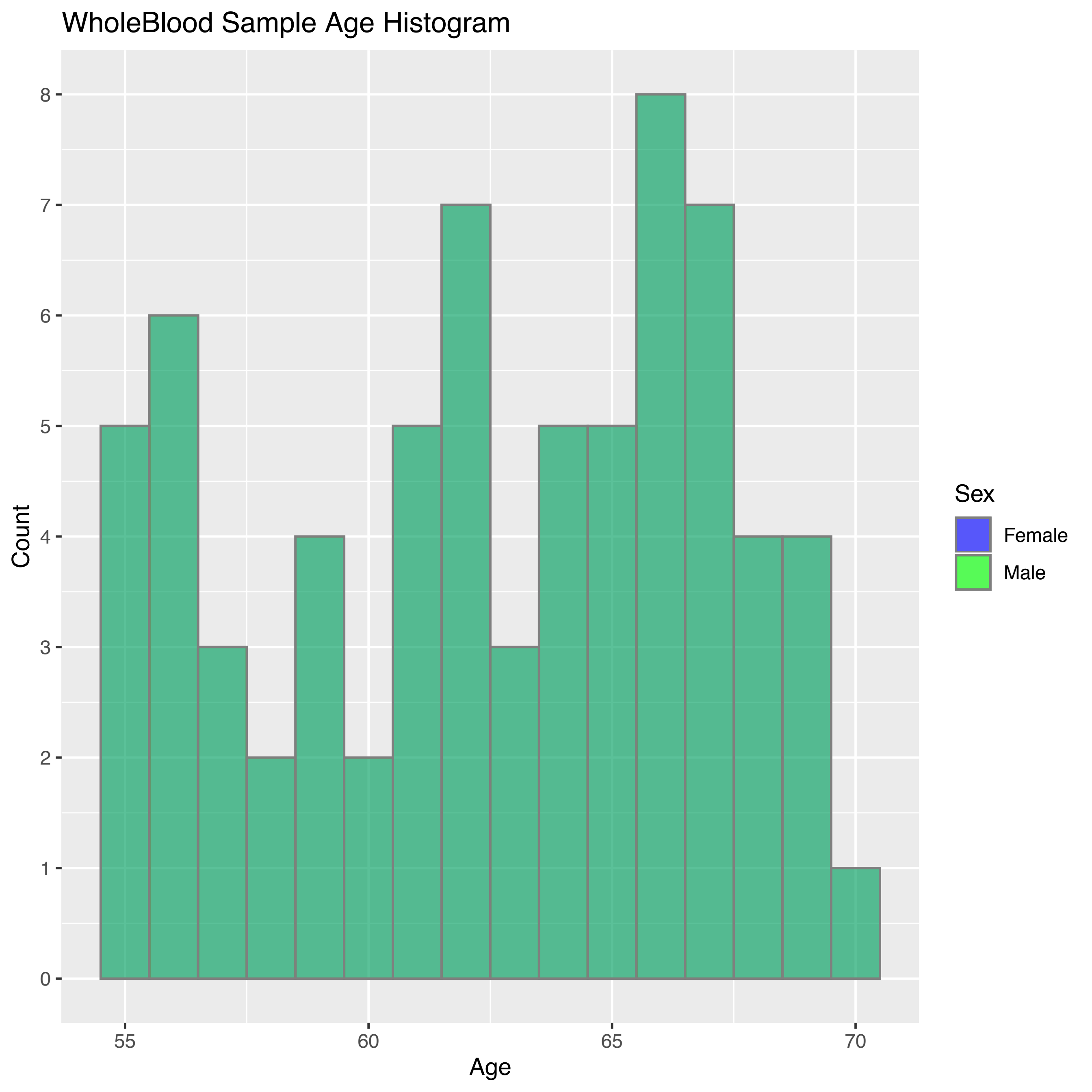

WholeBlood Sample Age Histogram
Count
Age
0
1
2
3
4
5
6
7
8
55
60
65
70
Sex
Female
Male

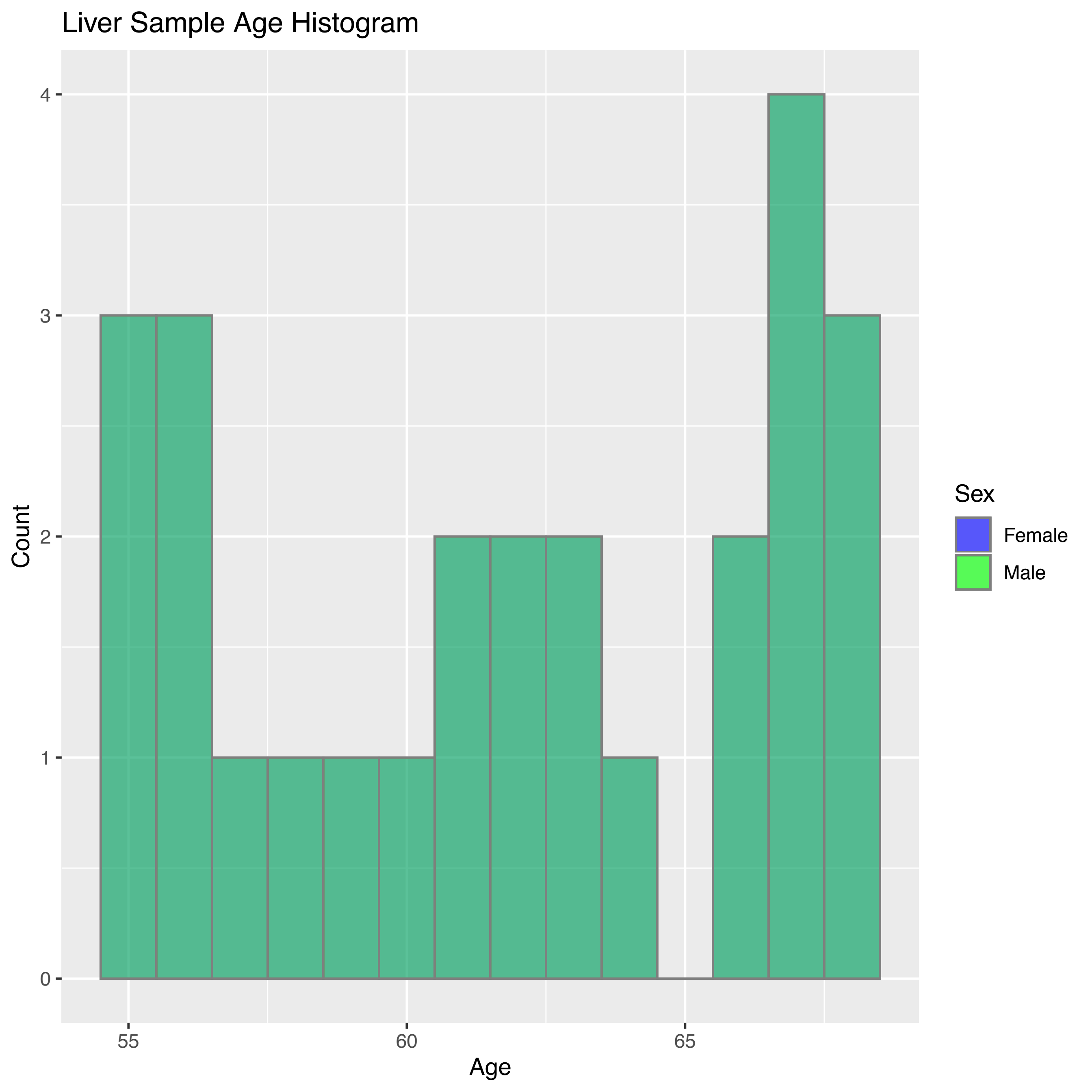
Liver Sample Age Histogram
Count
4
3
2
1
0
55
60
65
Age
Sex
Female
Male

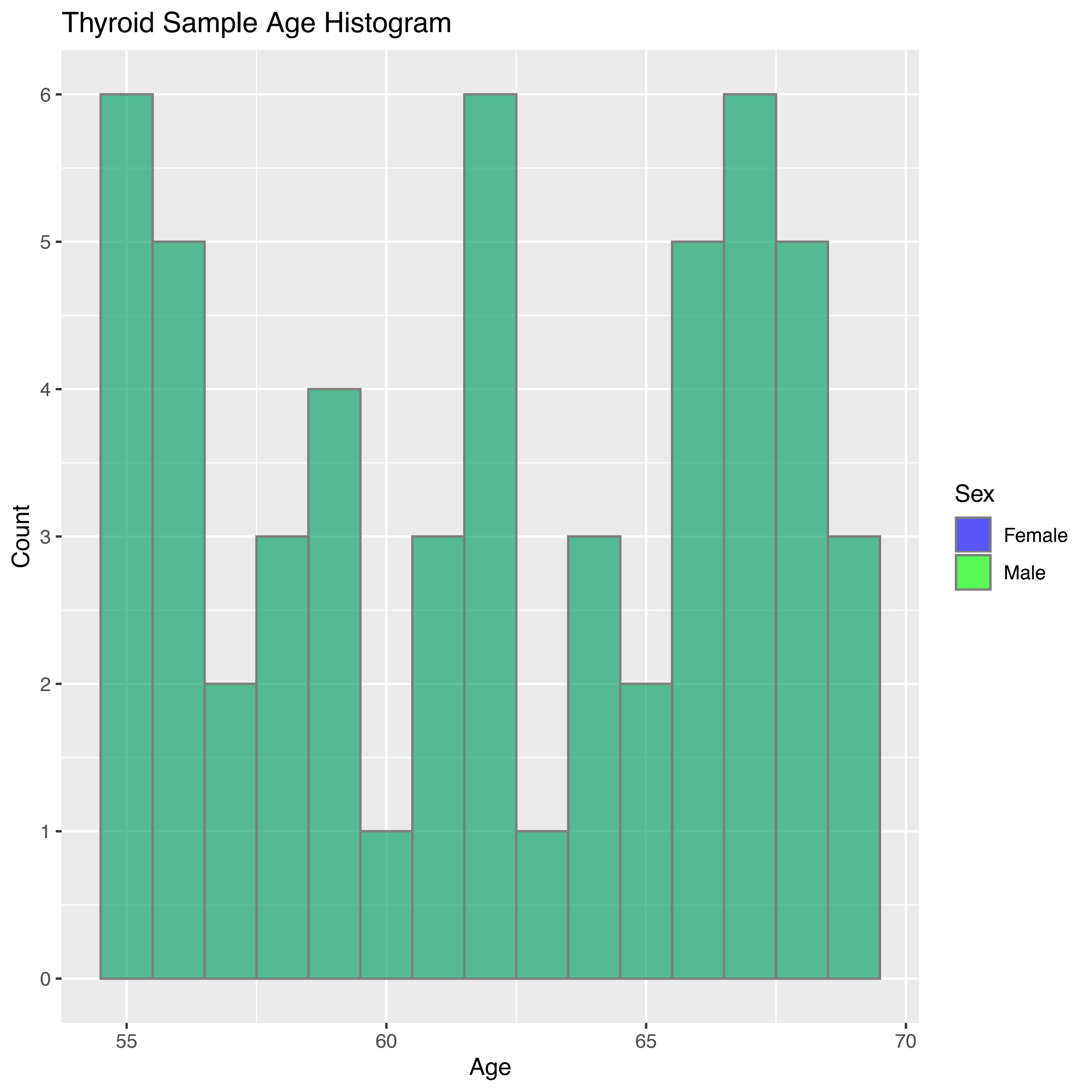

Thyroid Sample Age Histogram
Count
6
5
4
3
2
1
0
55
60
65
70
Age
Sex
Female
Male
