# Supplementary material for: Reference genome and transcriptome informed by the sex chromosome complement of the sample increase ability to detect sex differences in gene expression from RNA-Seq data
Source: Biol Sex Differ. 2020 Jul 21;11:42. doi: 10.1186/s13293-020-00312-9 (PMC7374973; doi:10.1186/s13293-020-00312-9)

A

All TISSUES aligned to STAR and default reference genome

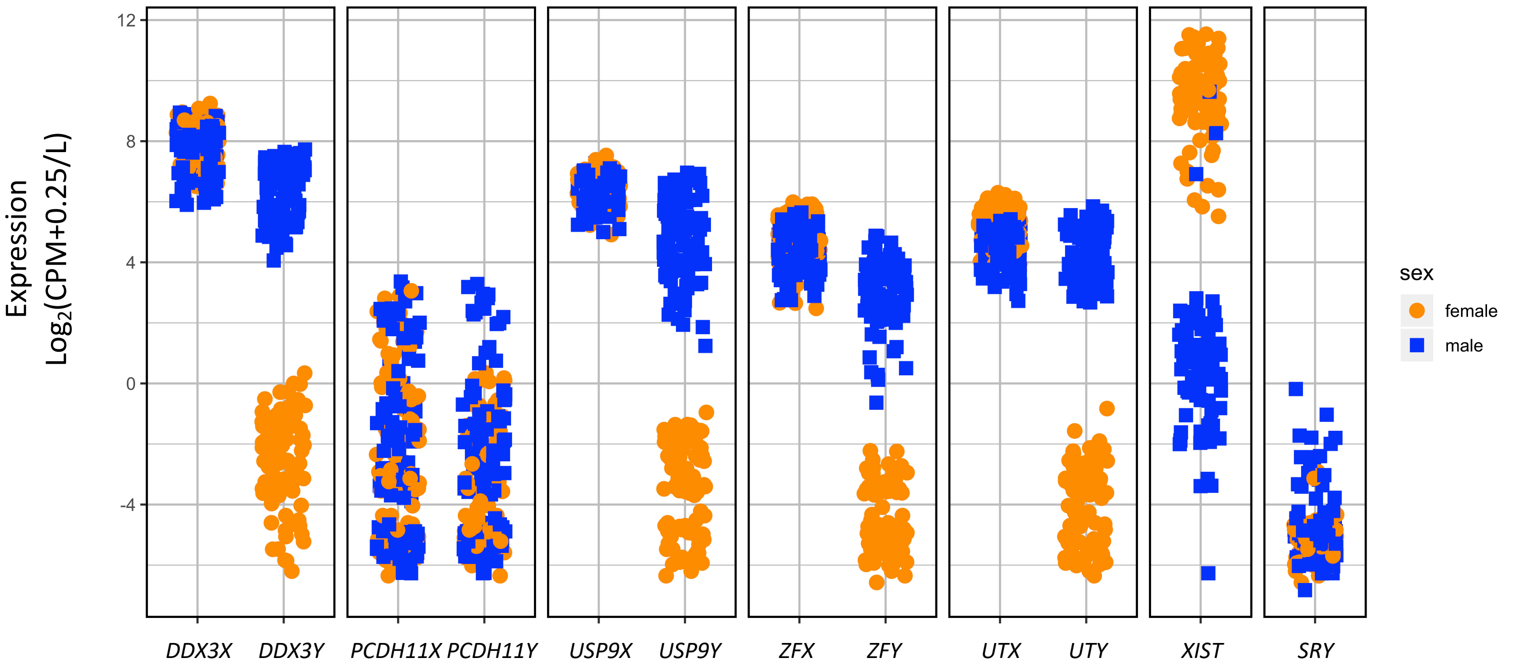

B

All TISSUES aligned to STAR and sex chromosome complement reference genome

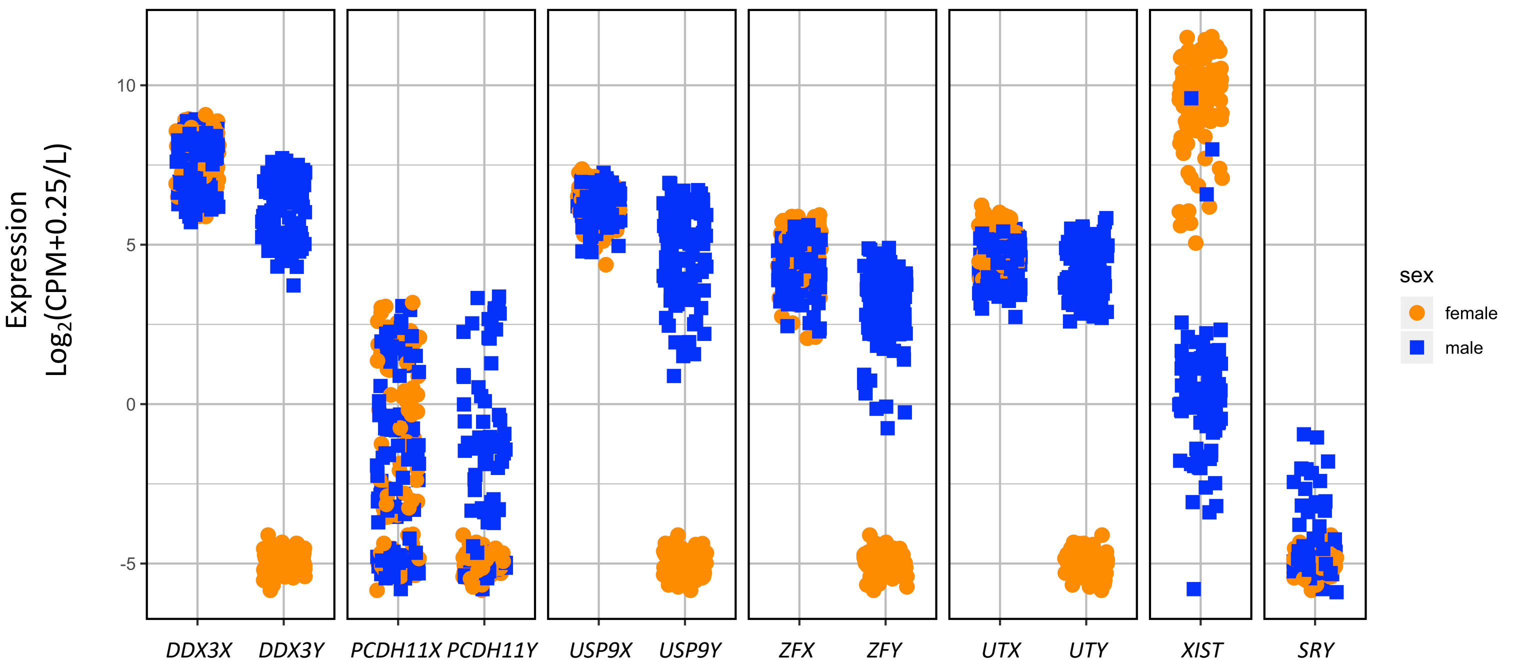

Supplement: Supplementary file 3 — Additional file 3: Genetic sex of RNA-Seq samples when aligned using STAR. Gene expression log2(CPM + 0.25/L) for select XY homologous genes (DDX3X/Y, PCDH11X/Y, USP9X/Y, ZFX/Y, UTX/Y) and XIST and SRY when reads were aligned to a default reference genome A), and for B) when reads were aligned to a sex chromosome complement informed reference using STAR. Male XY whole blood, brain cortex, breast, liver, and thyroid samples are shown in blue squares and female XX in orange circles. [file 13293_2020_312_MOESM3_ESM.pdf]
