# Supplementary material for: Reference genome and transcriptome informed by the sex chromosome complement of the sample increase ability to detect sex differences in gene expression from RNA-Seq data
Source: Biol Sex Differ. 2020 Jul 21;11:42. doi: 10.1186/s13293-020-00312-9 (PMC7374973; doi:10.1186/s13293-020-00312-9)

A BLOOD aligned to HISAT and default reference genome

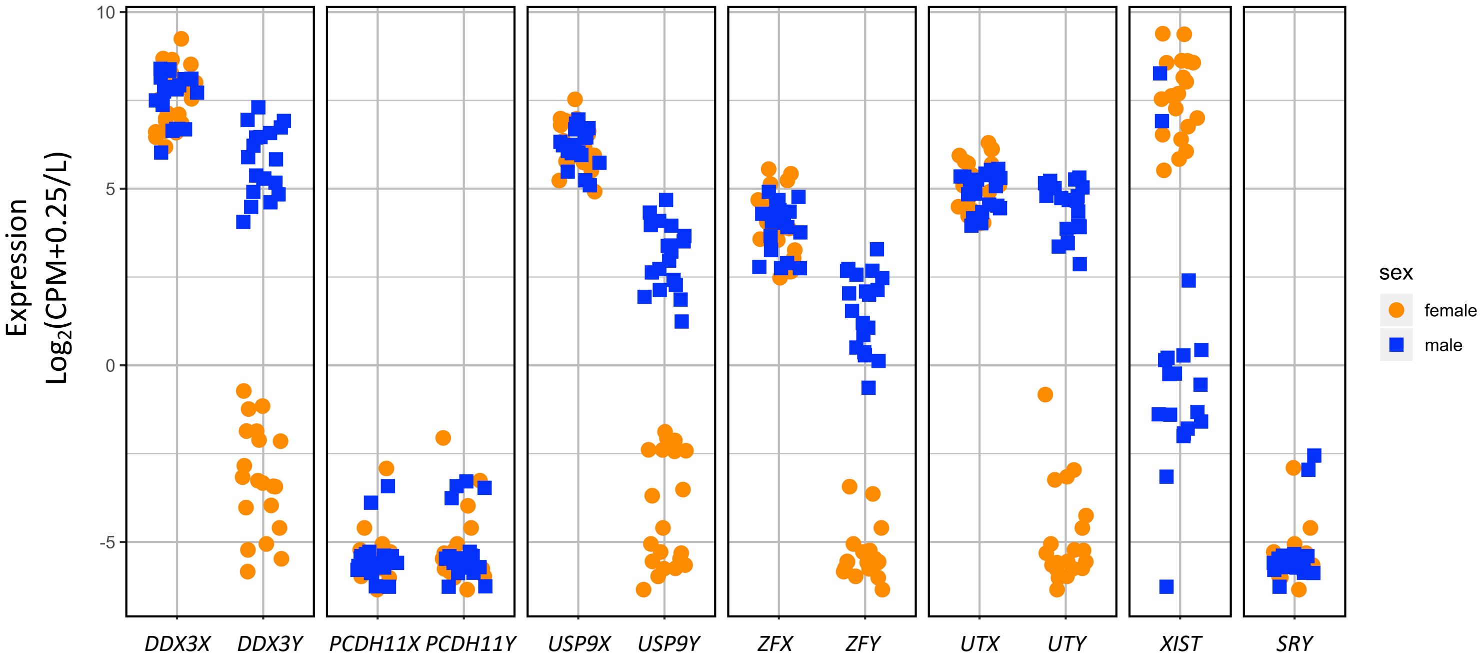

B BLOOD aligned to HISAT and sex chromosome complement reference genome

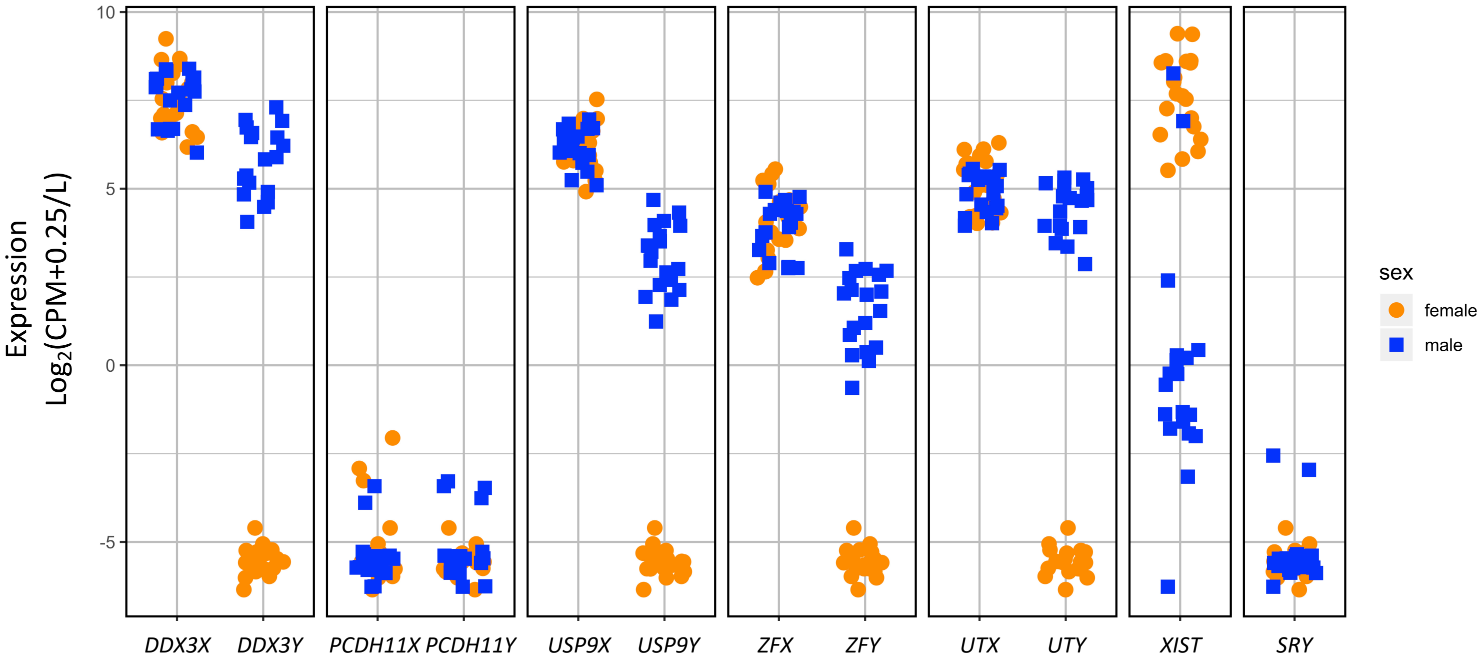

C BLOOD aligned to STAR and default reference genome

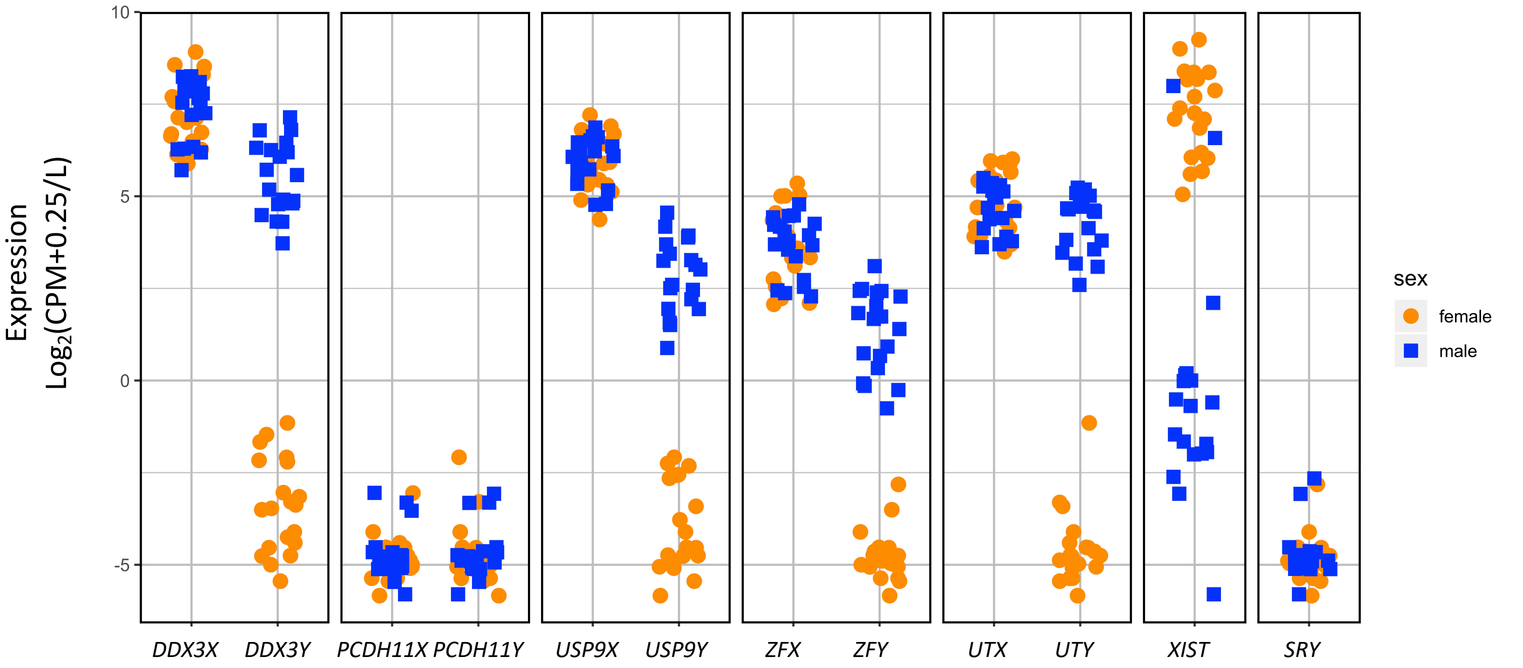

D BLOOD aligned to STAR and sex chromosome complement reference genome

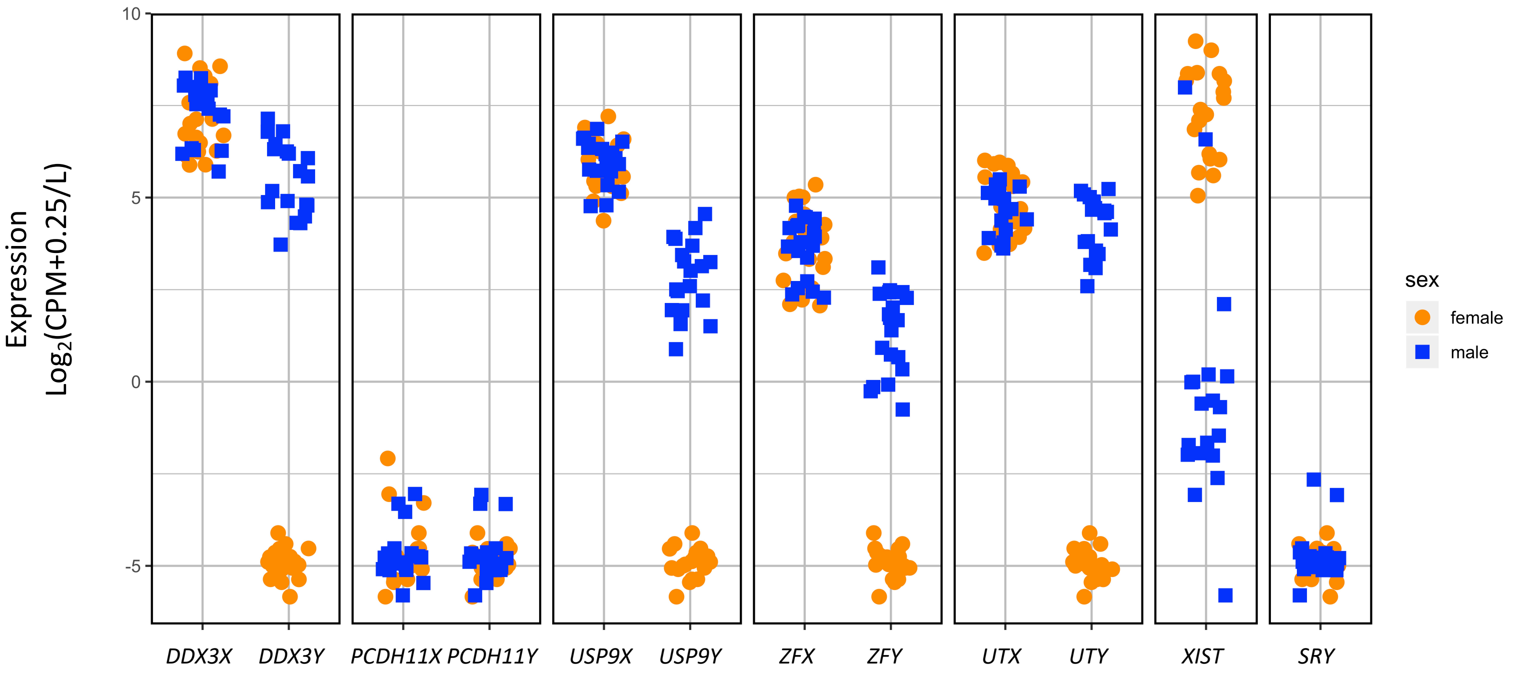

E

BRAIN CORTEX aligned to HISAT and default reference genome

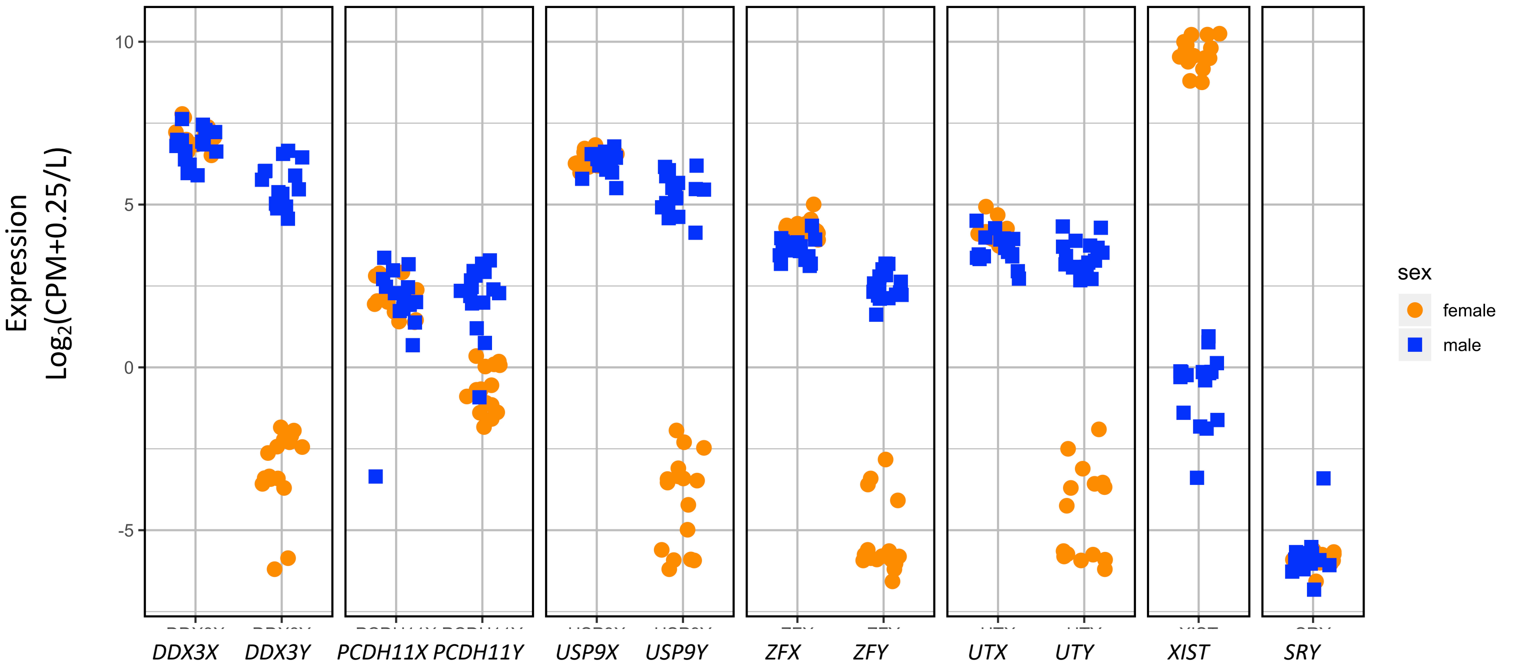

F

BRAIN CORTEX aligned to HISAT and sex chromosome complement reference genome

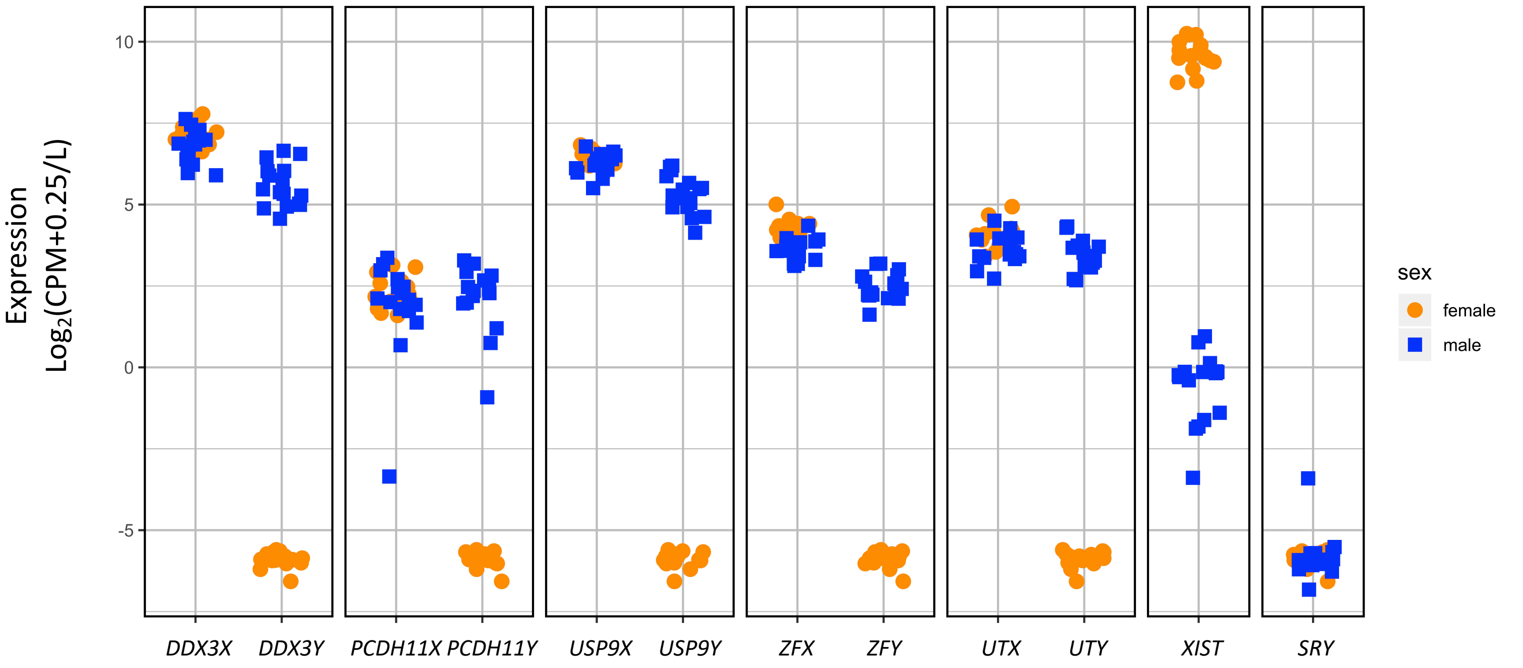

G BRAIN CORTEX aligned to STAR and default reference genome

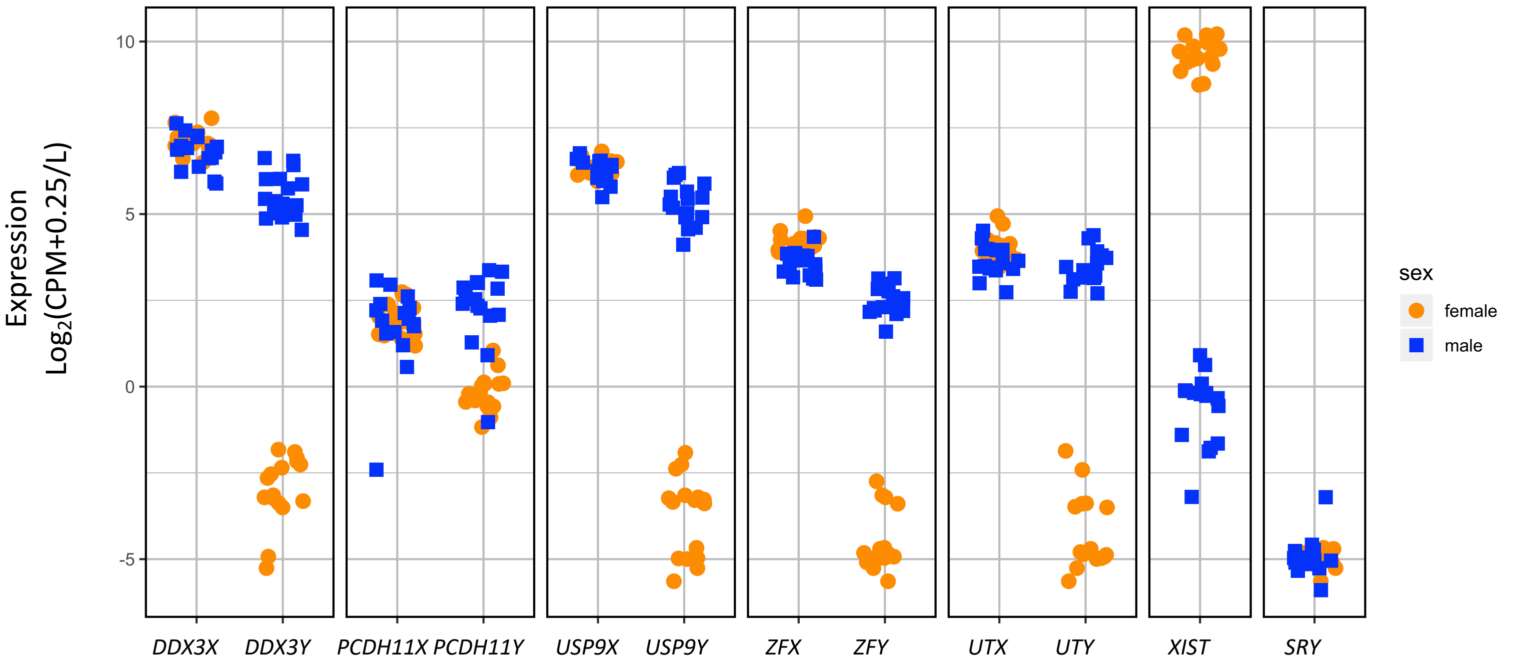

H BRAIN CORTEX aligned to STAR and sex chromosome complement reference genome

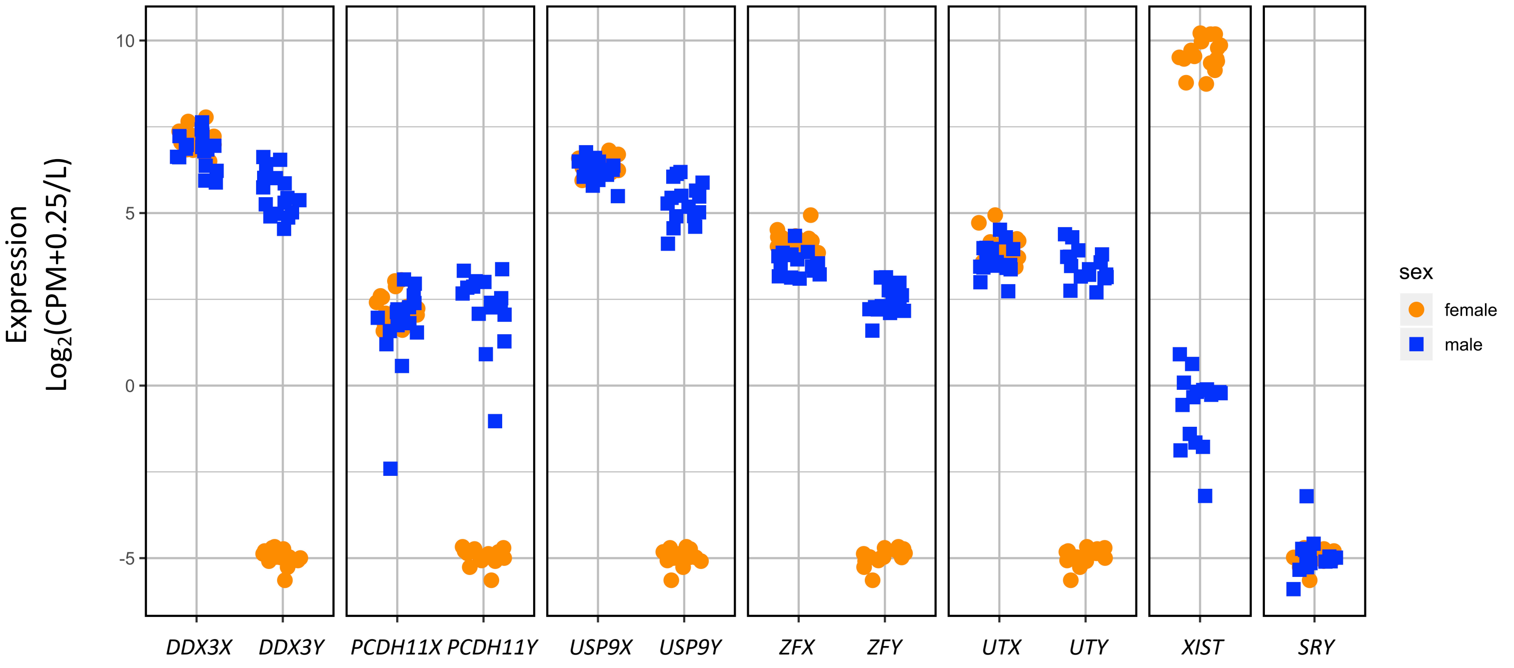

I

BREAST aligned to HISAT and default reference genome

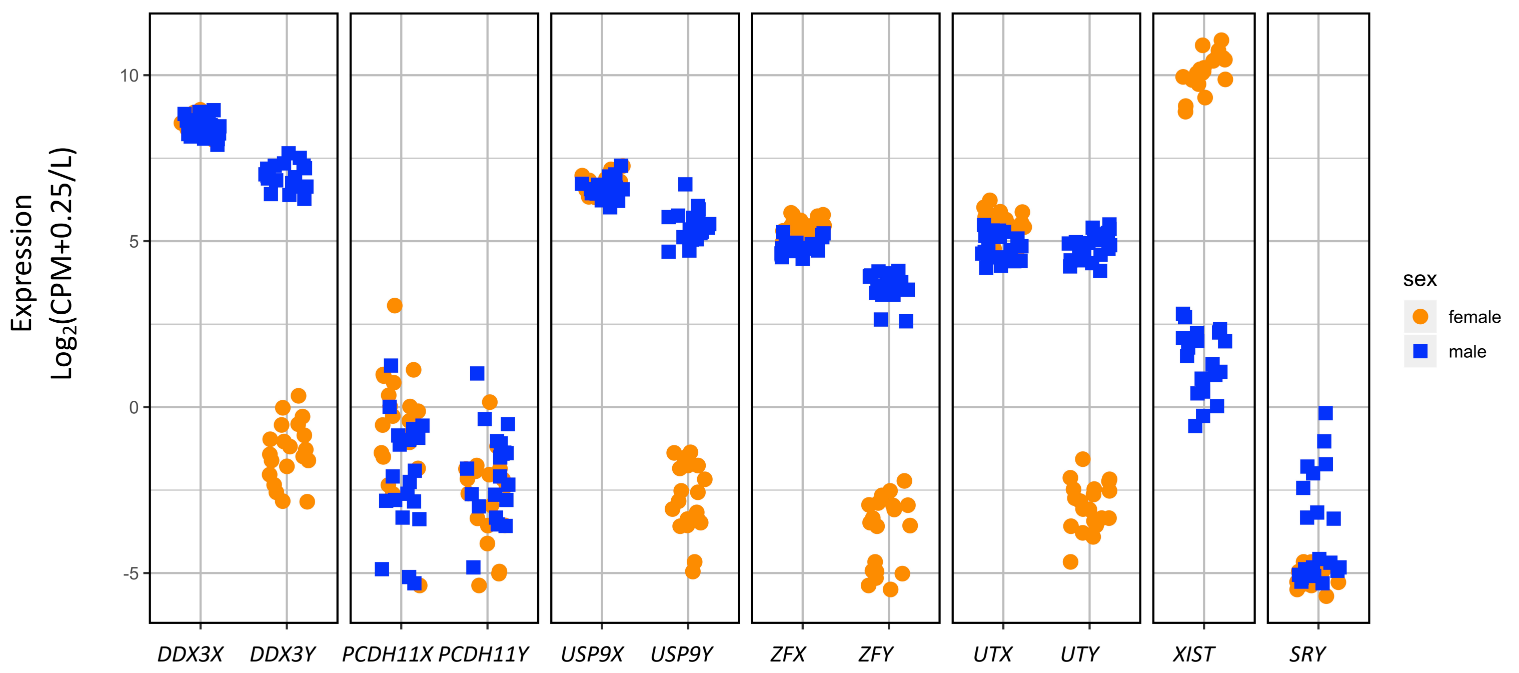

J

BREAST aligned to HISAT and sex chromosome complement reference genome

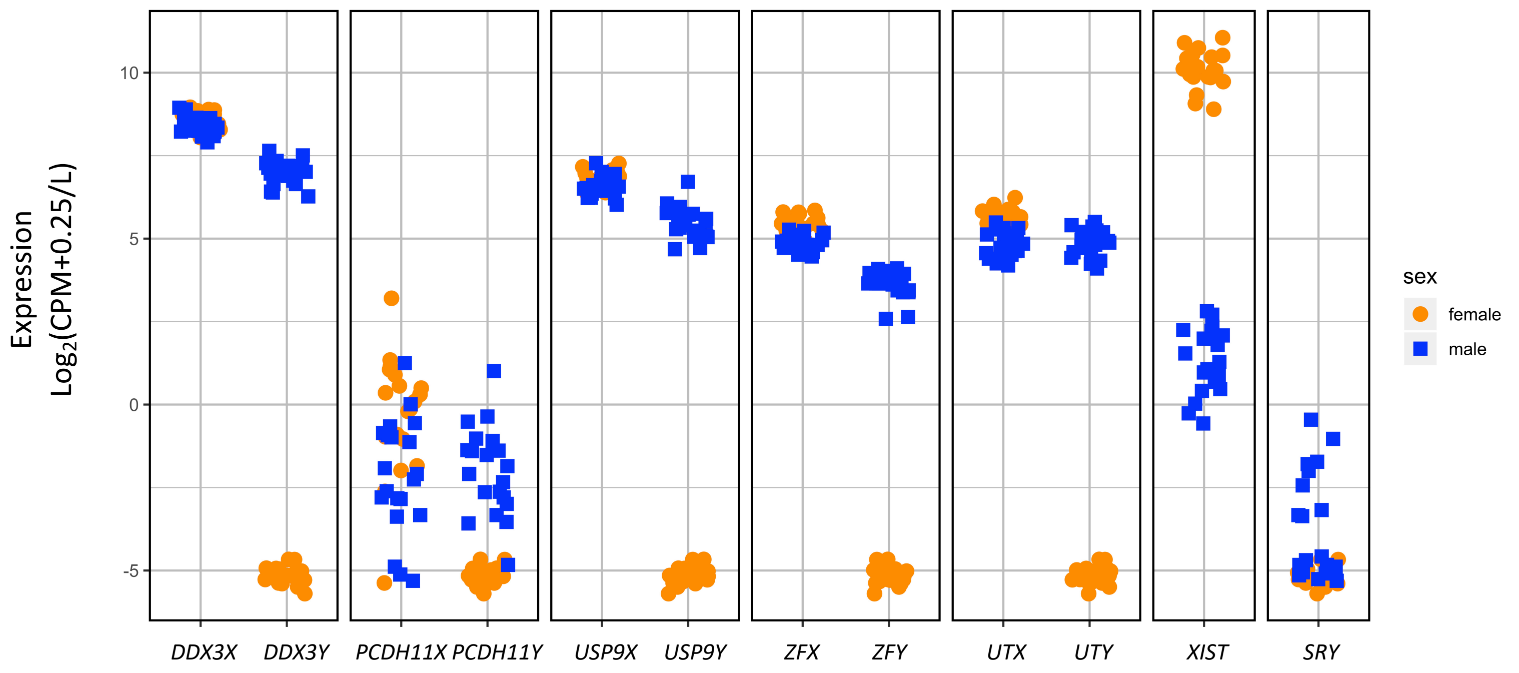

K

BREAST aligned to STAR and default reference genome

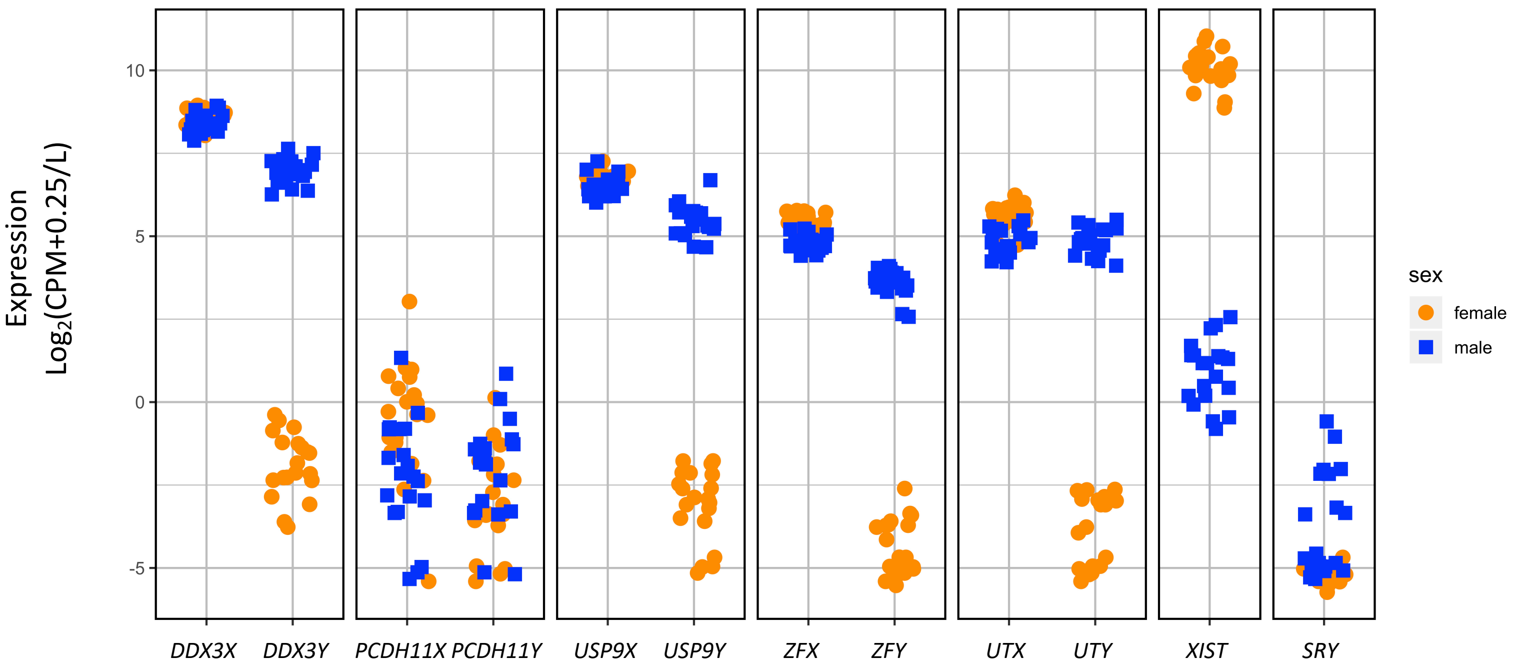

L

BREAST aligned to STAR and sex chromosome complement reference genome

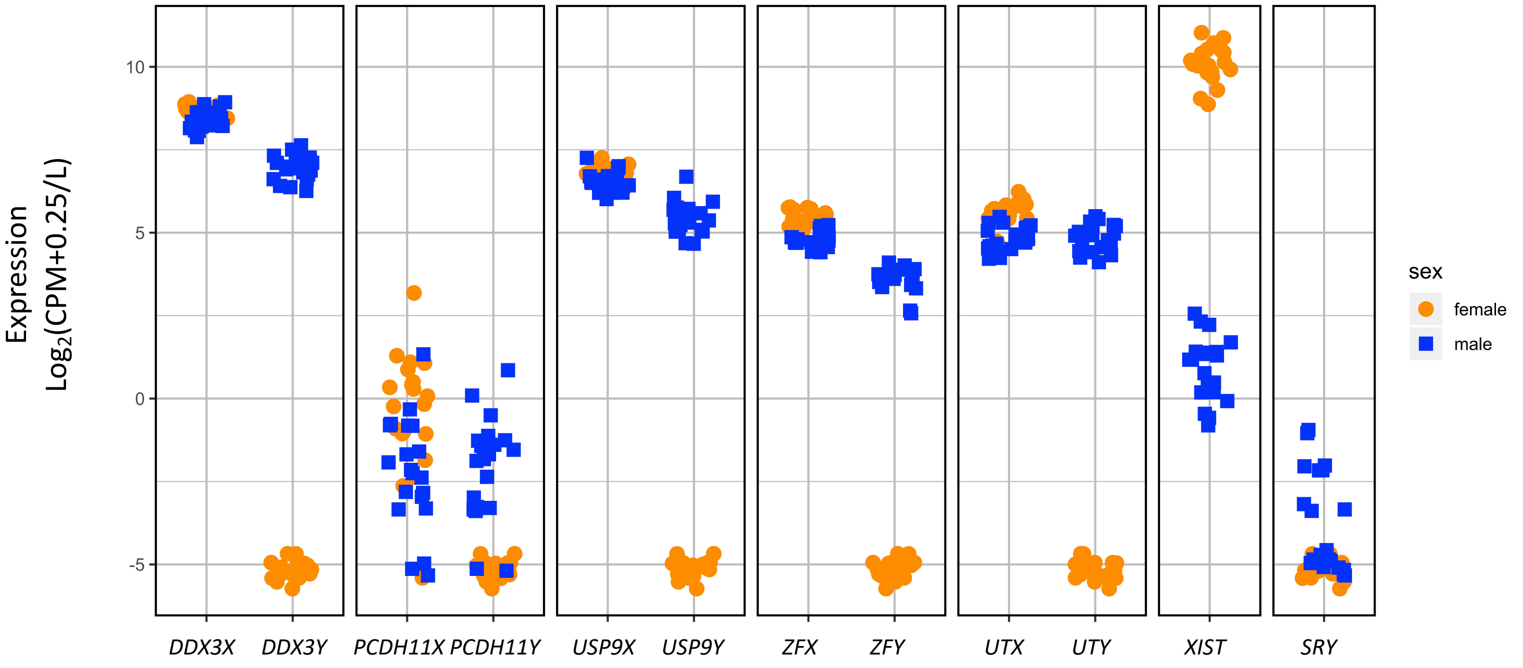

M LIVER aligned to HISAT and default reference genome

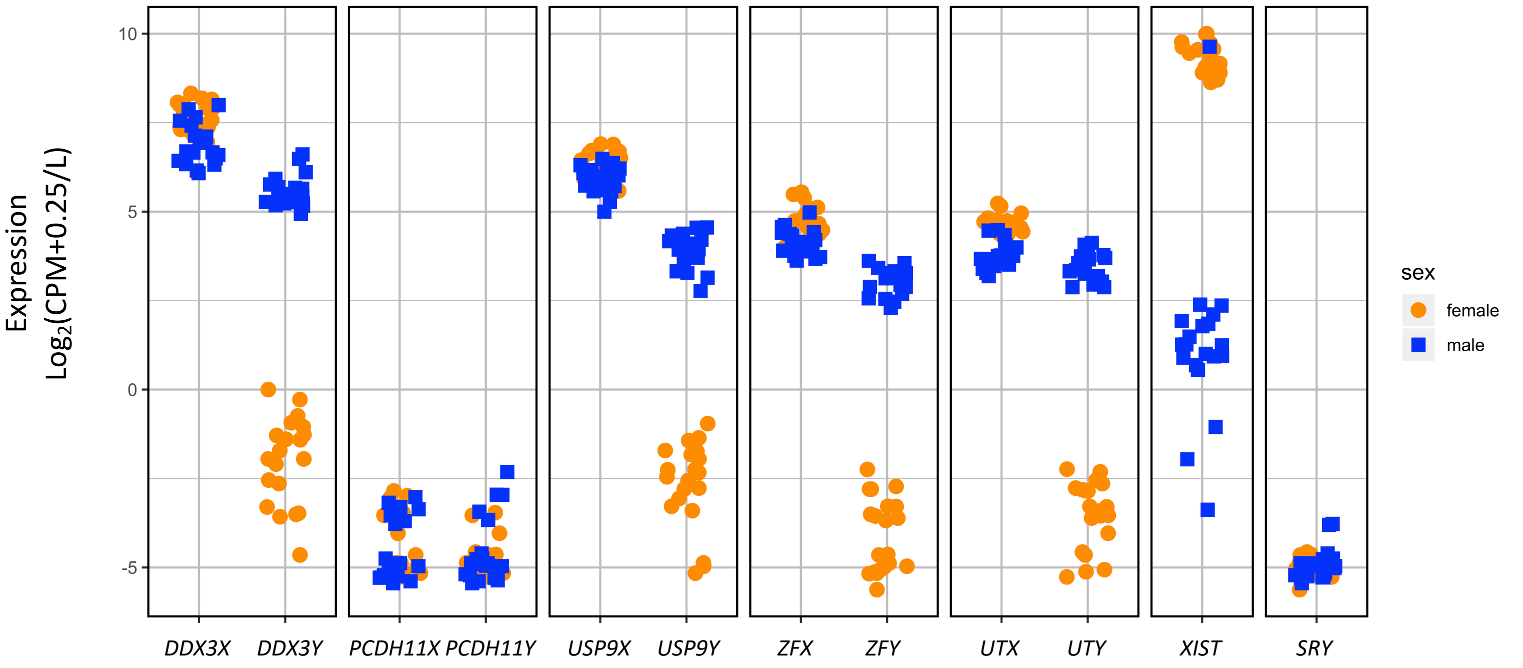

N LIVER aligned to HISAT and sex chromosome complement reference genome

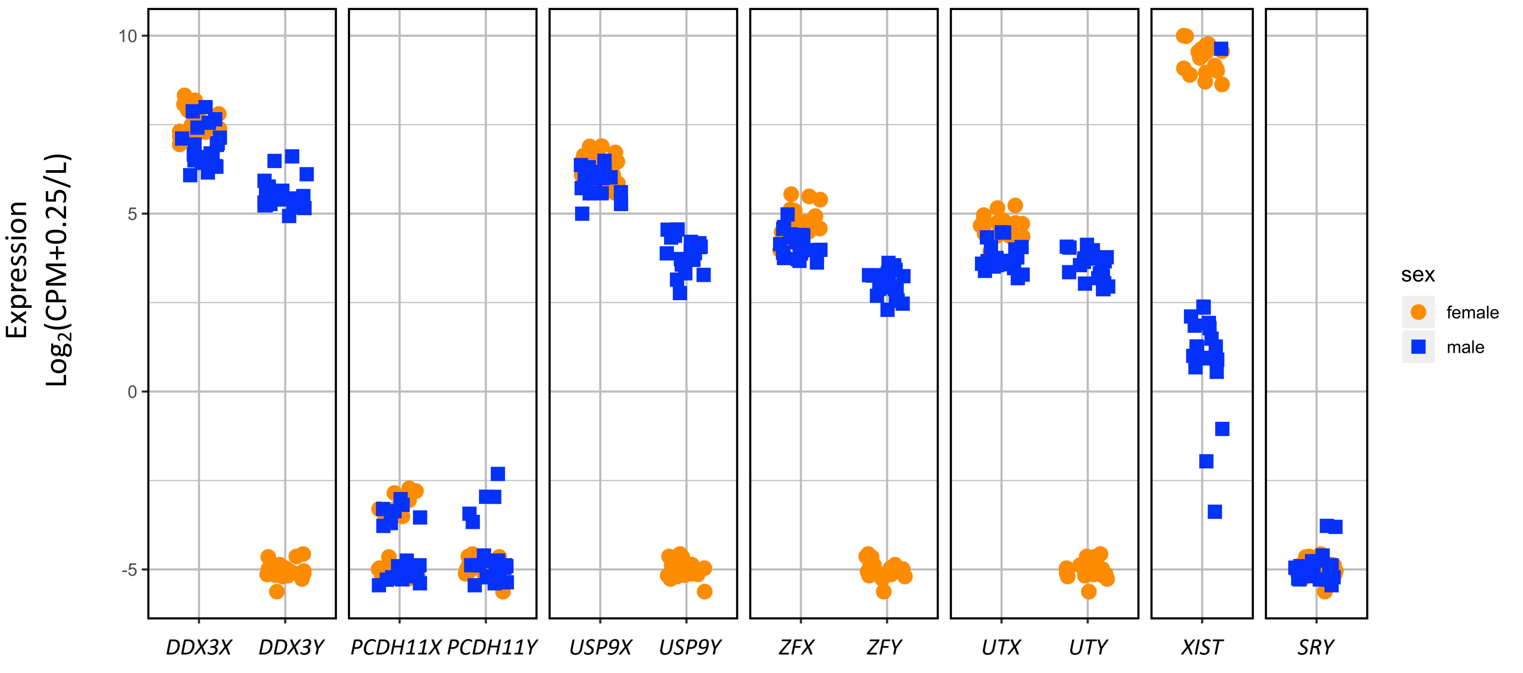

O LIVER aligned to STAR and default reference genome

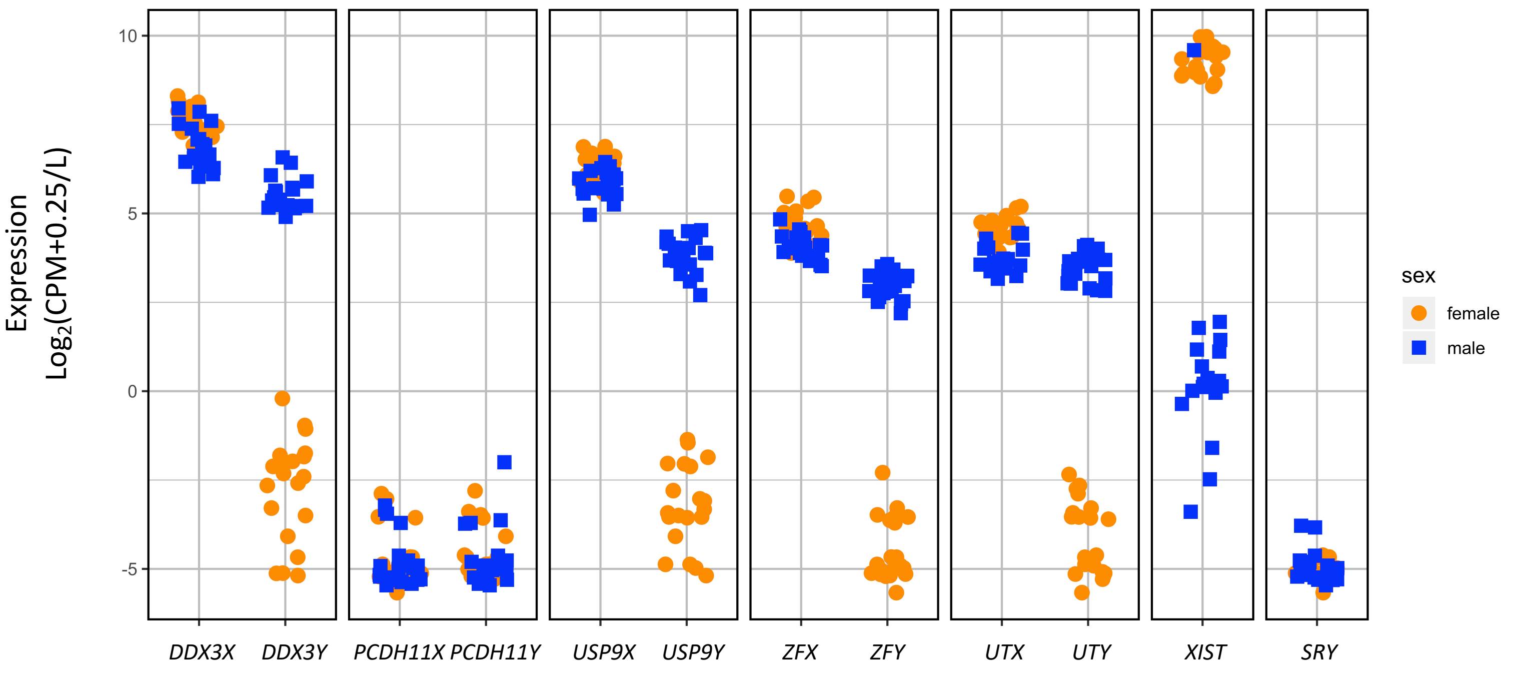

P LIVER aligned to STAR and sex chromosome complement reference genome

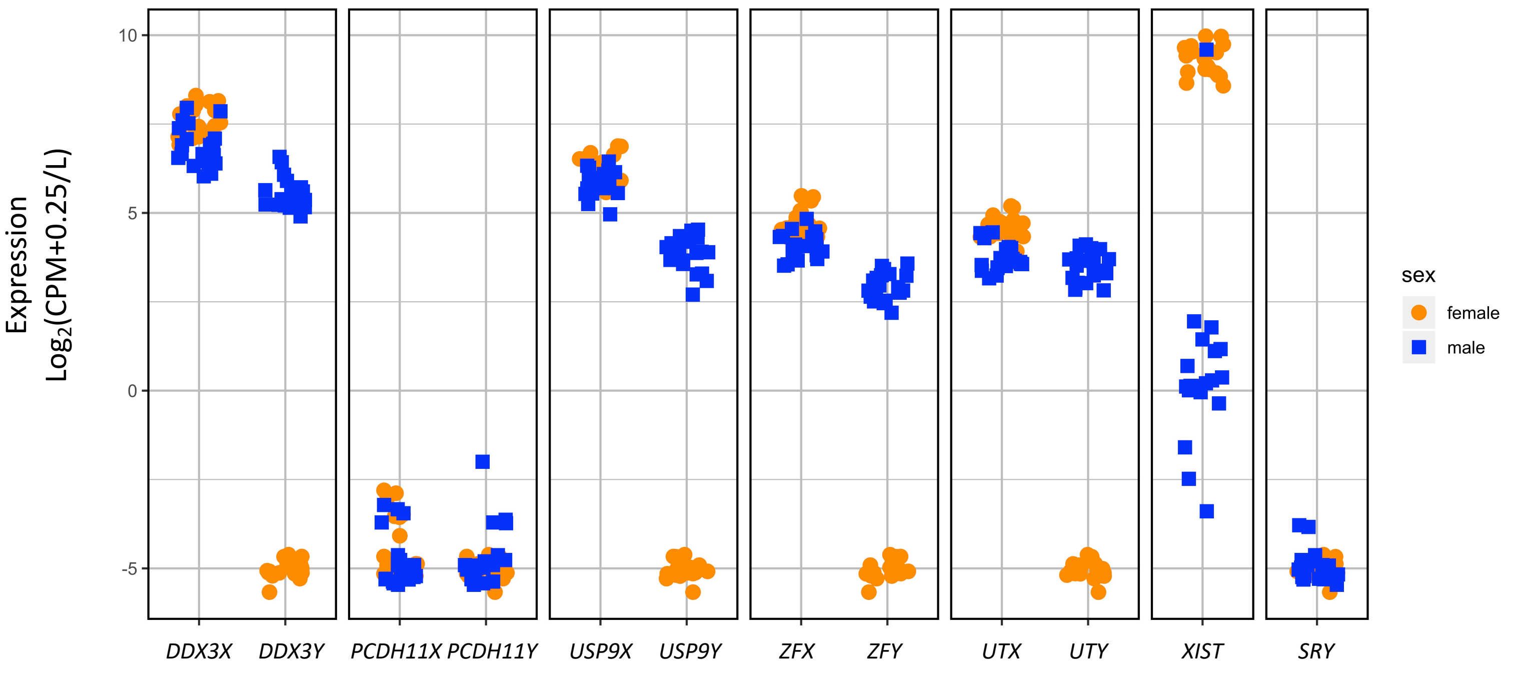

Q THYROID aligned to HISAT and default reference genome

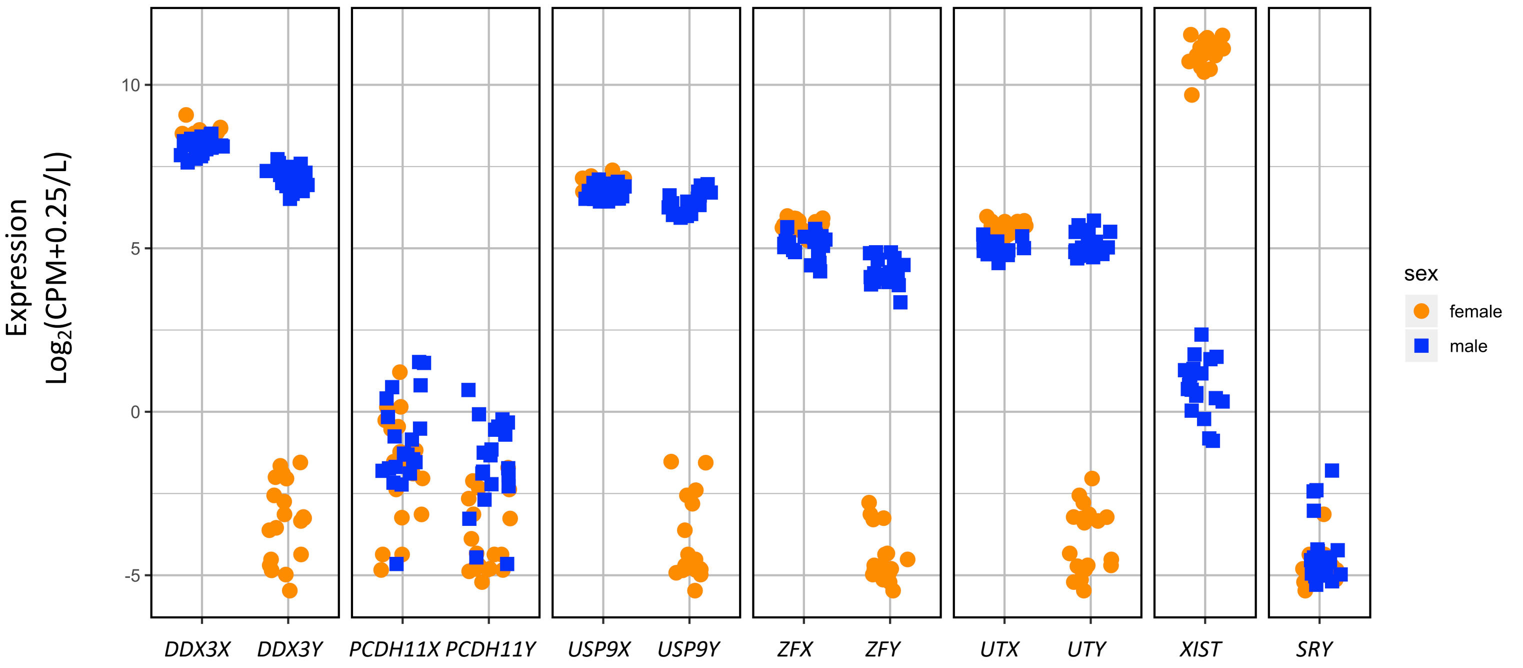

R THYROID aligned to HISAT and sex chromosome complement reference genome

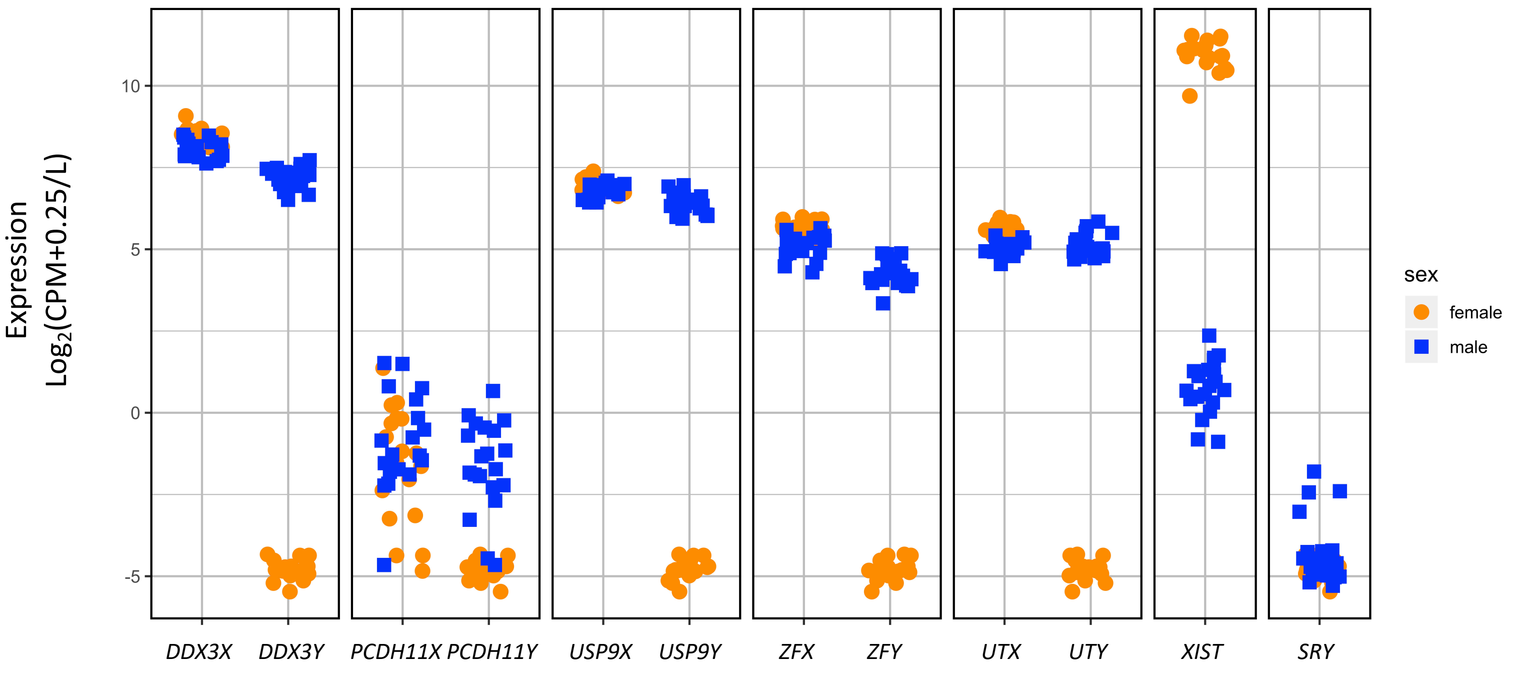

S THYROID aligned to STAR and default reference genome

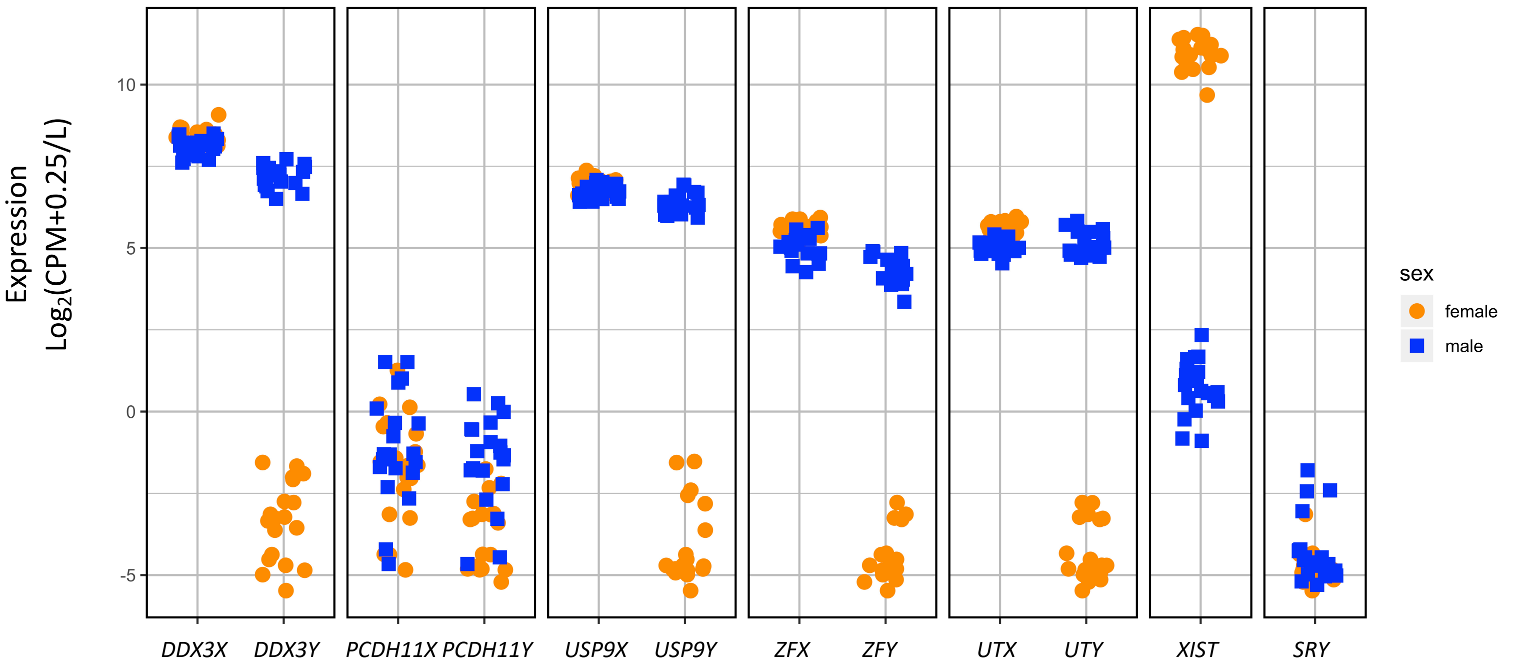

T THYROID aligned to STAR and sex chromosome complement reference genome

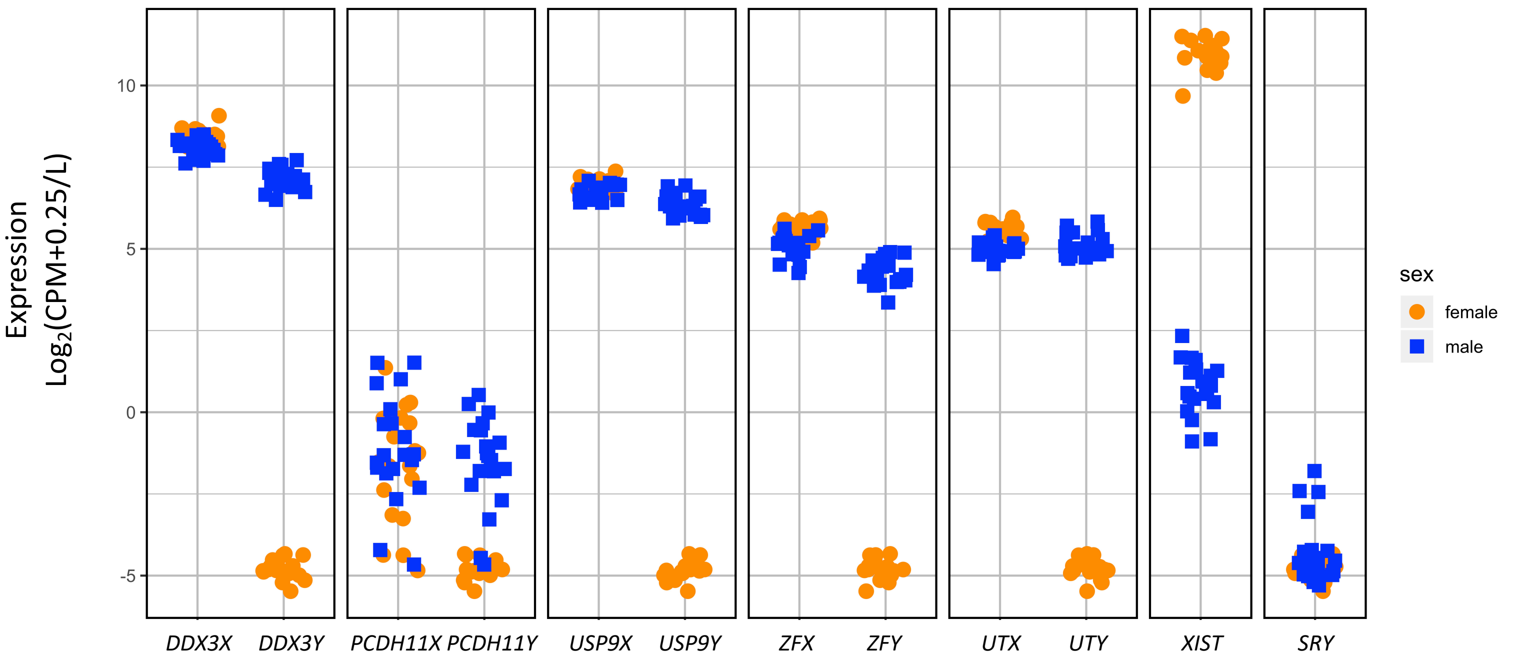

Supplement: Supplementary file 4 — Additional file 4: Genetic sex of RNA-Seq samples per tissue. Gene expression log2(CPM + 0.25/L) for select XY homologous genes (DDX3X/Y, PCDH11X/Y, USP9X/Y, ZFX/Y, UTX/Y) and XIST and SRY when reads were aligned to a default reference genome A), and for B) when reads were aligned to a sex chromosome complement informed reference using HISAT and C) and D), for when the reads were aligned using STAR. Male XY whole blood, brain cortex, breast, liver, and thyroid samples are shown in blue squares and female XX in orange circles. [file 13293_2020_312_MOESM4_ESM.pdf]
