# Supplementary material for: Reference genome and transcriptome informed by the sex chromosome complement of the sample increase ability to detect sex differences in gene expression from RNA-Seq data
Source: Biol Sex Differ. 2020 Jul 21;11:42. doi: 10.1186/s13293-020-00312-9 (PMC7374973; doi:10.1186/s13293-020-00312-9)

A) Blood PC1 & PC2

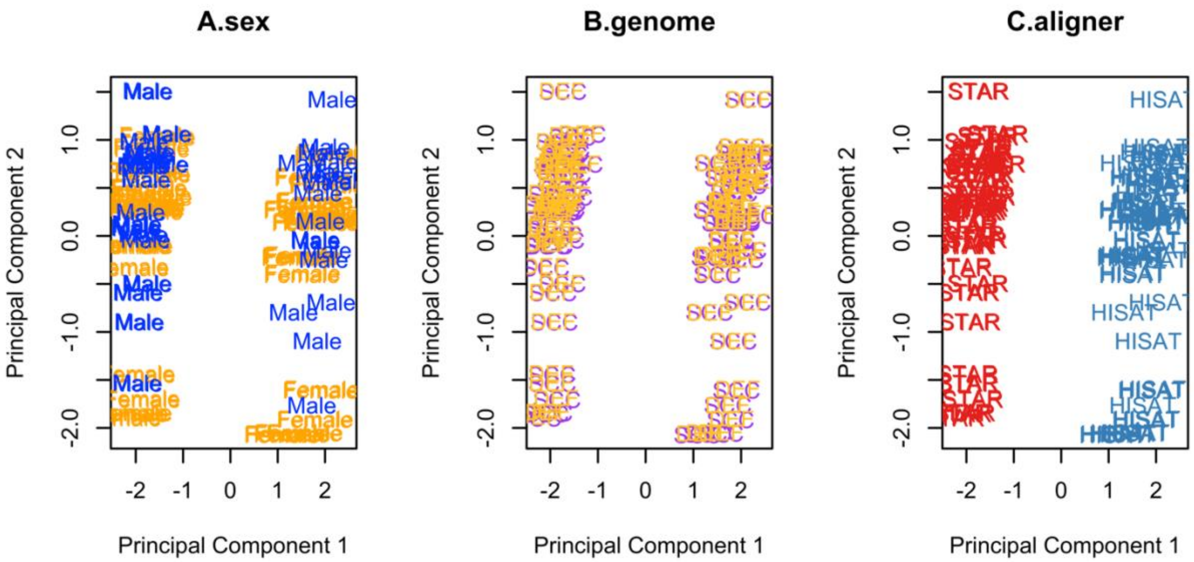

B) Blood PC2 & PC3

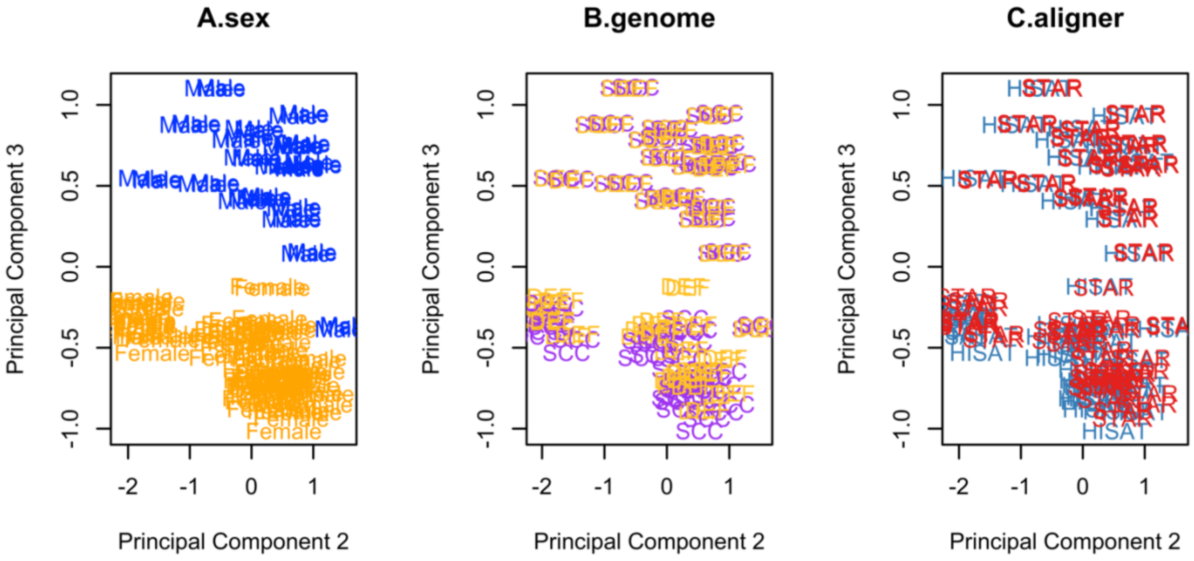

C) Brain cortex PC1 & PC2

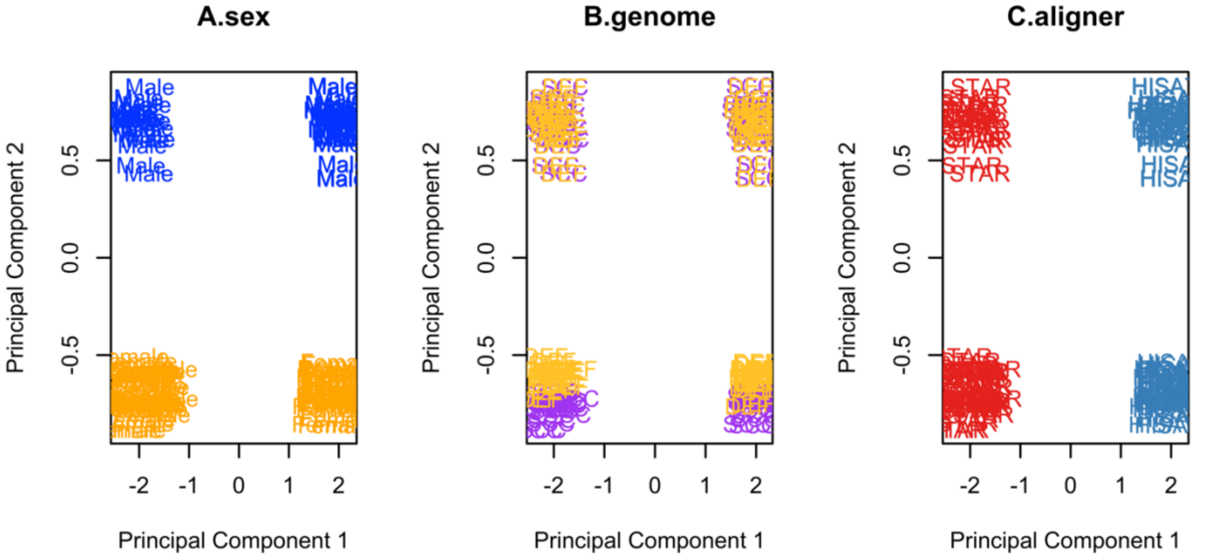

D) Brain cortex PC2 & PC3

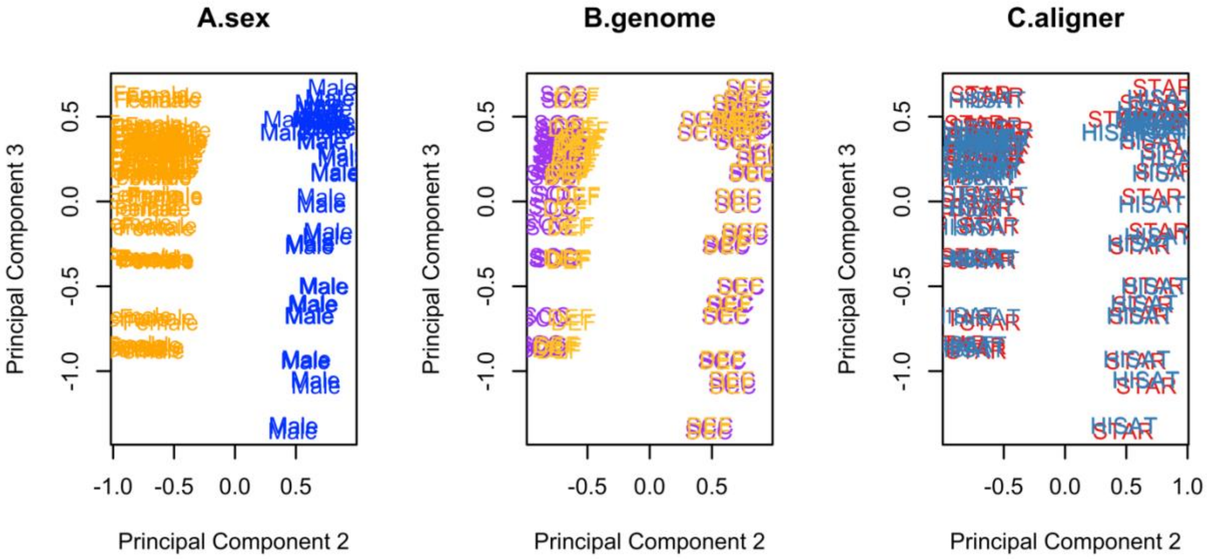

E) Breast PC1 & PC2

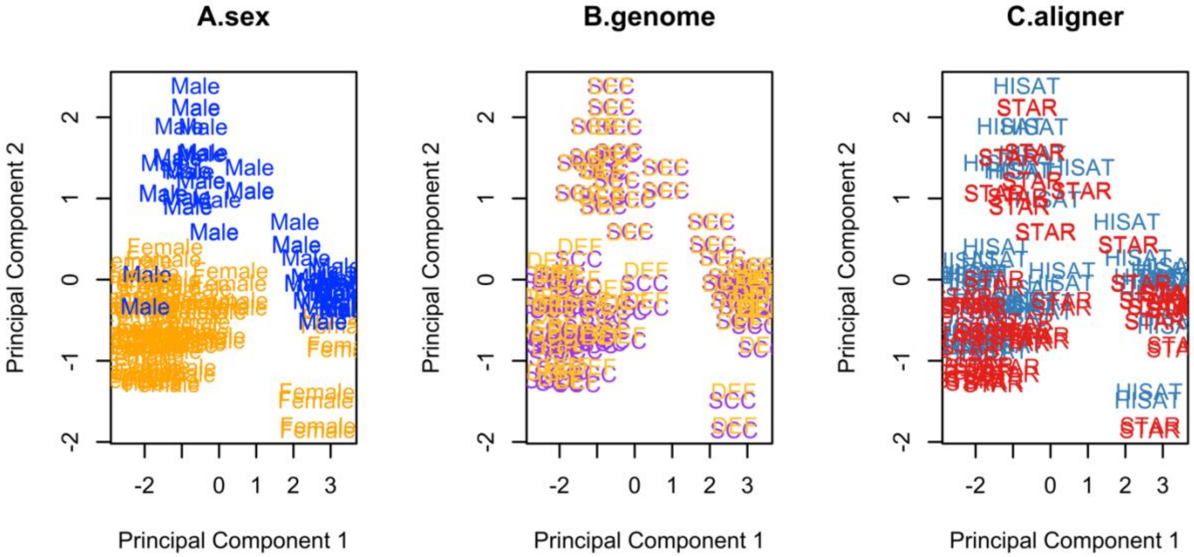

F) Breast PC2 & PC3

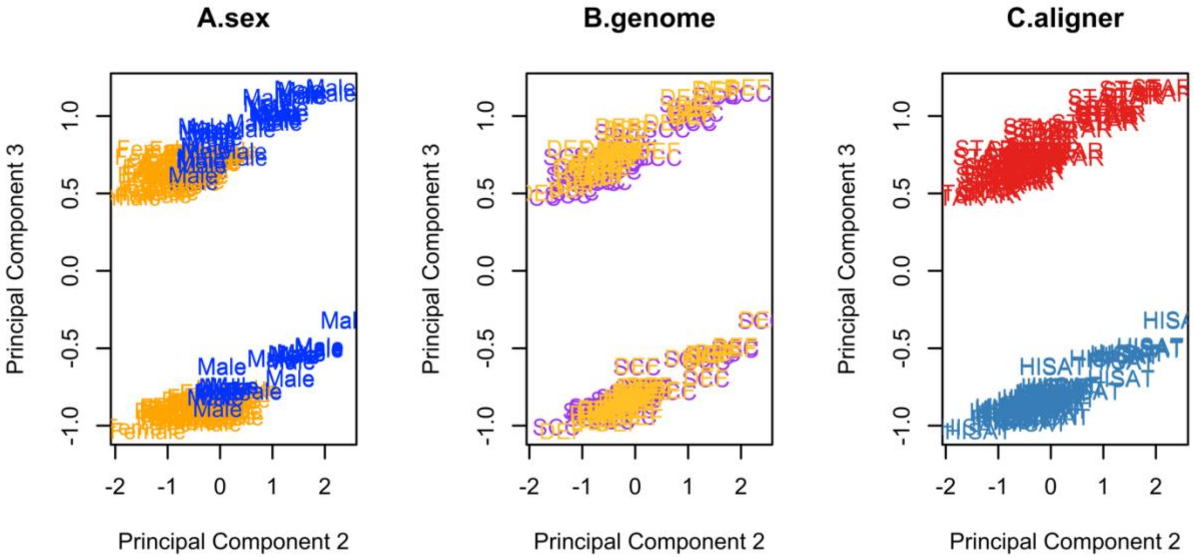

G) Liver PC1 & PC2

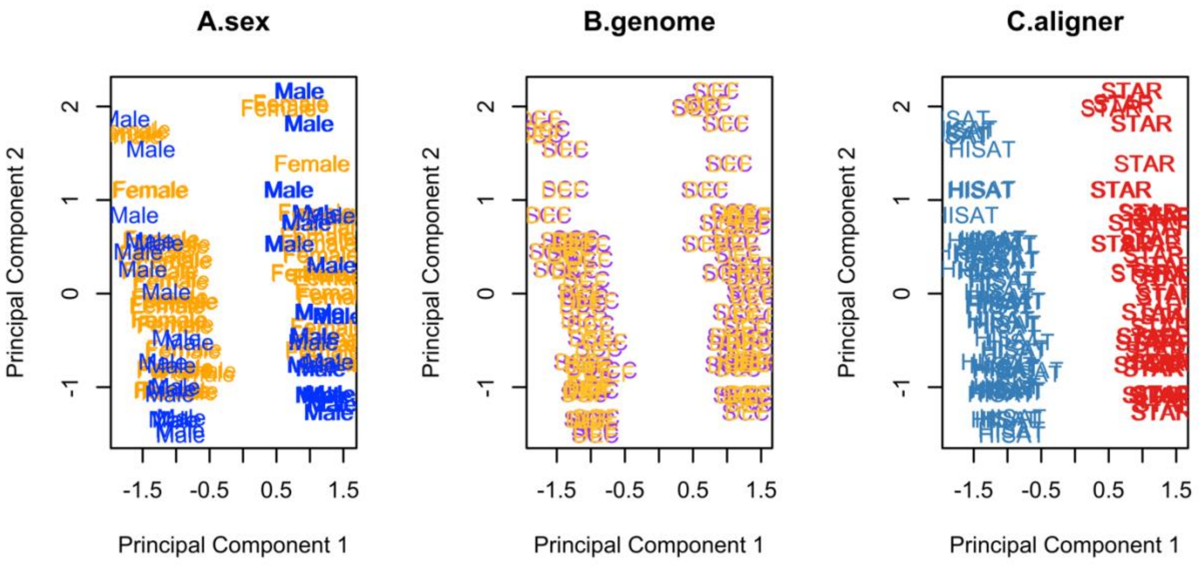

H) Liver PC2 & PC3

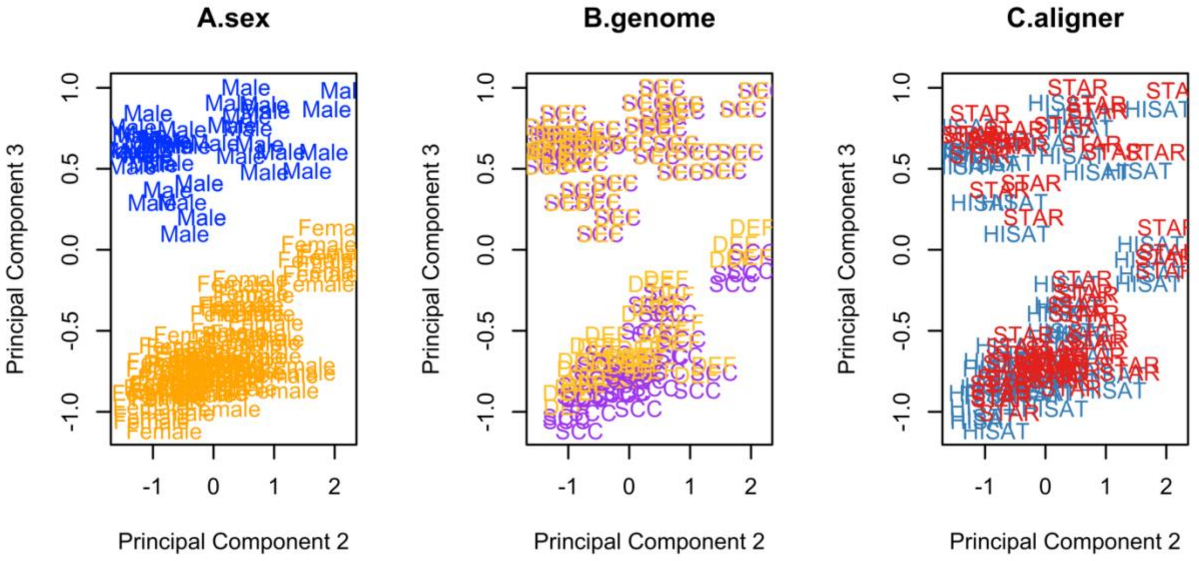

I) Thyroid PC1 & PC2

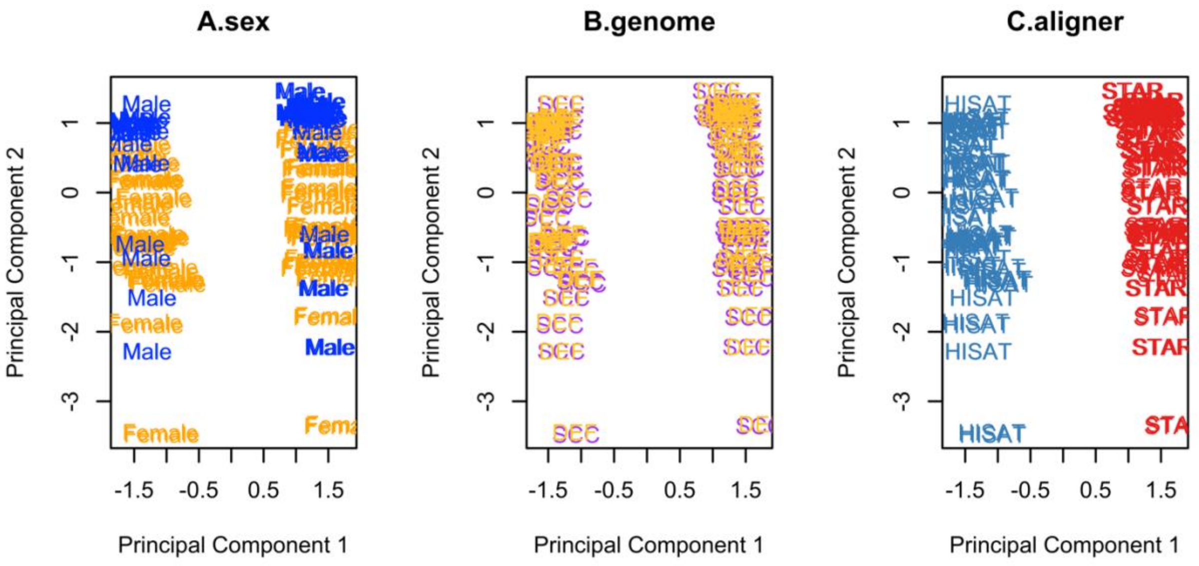

J) Thyroid PC2 & PC3

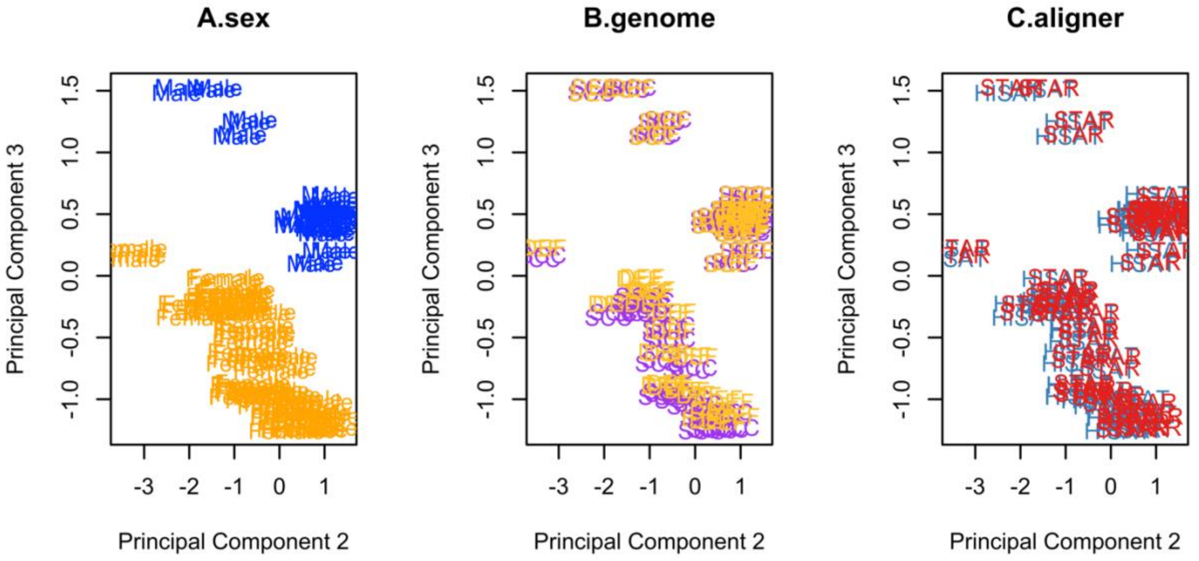

Supplement: Supplementary file 6 — Additional file 6: Multidimensional Scaling plots. We investigated multidimensional scaling for all shared common variable genes for dimensions 1 and 2, and for dimensions 2 and 3 in each tissue. The most variation in each tissue is explained by the aligner C.aligner. The second most variation in each tissue is explained by the sex of the sample A.sex. [file 13293_2020_312_MOESM6_ESM.pdf]
