# Supplementary figures and images for: Reference genome and transcriptome informed by the sex chromosome complement of the sample increase ability to detect sex differences in gene expression from RNA-Seq data
Source: Biol Sex Differ. 2020 Jul 21;11:42. doi: 10.1186/s13293-020-00312-9 (PMC7374973; doi:10.1186/s13293-020-00312-9)

HISAT mapped reads

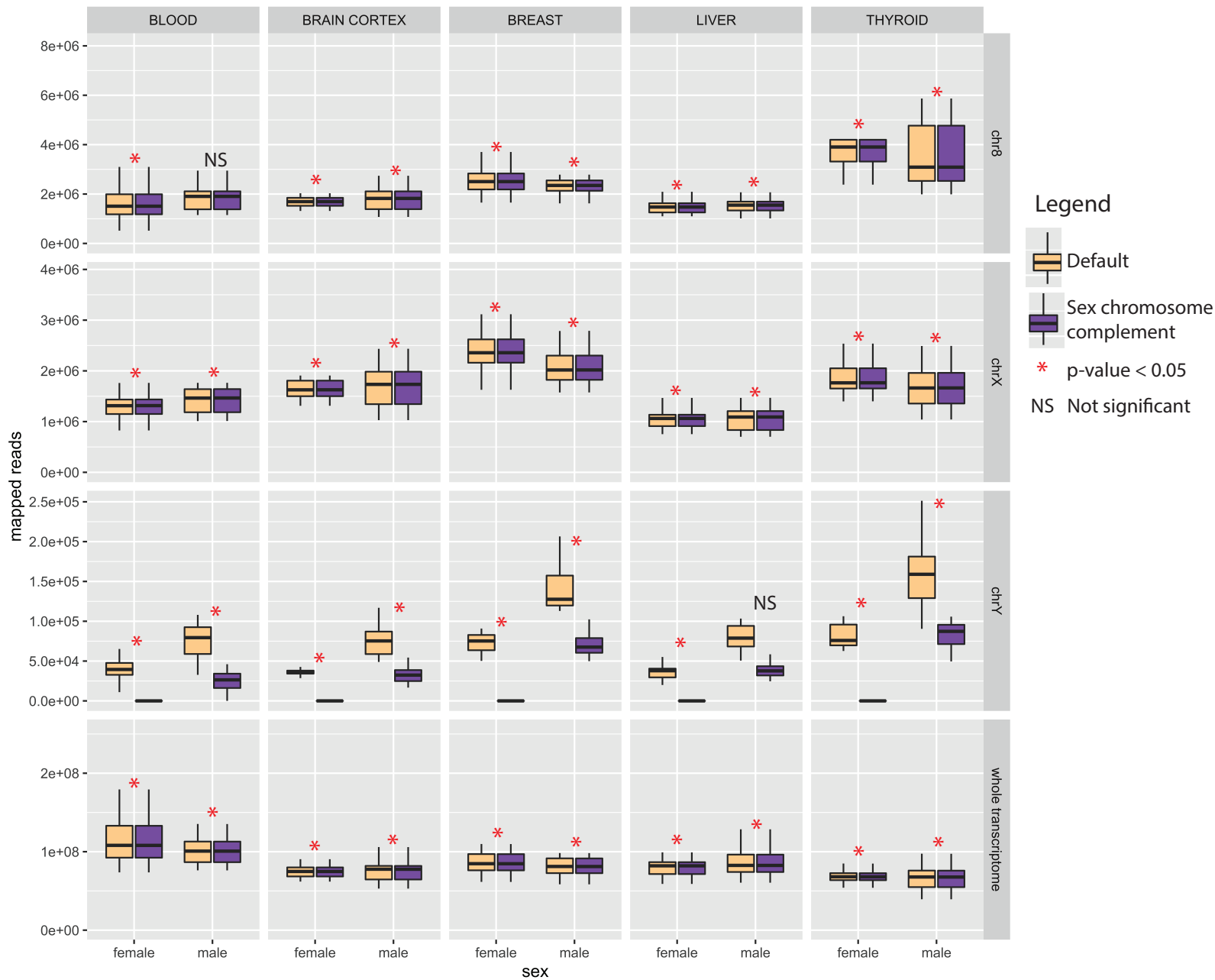

Supplement: Supplementary file 7 — Additional file 7: HISAT mapped reads bar plot. Mean difference in expression for average total reads mapped for each tissue and each sex when aligned to a sex chromosome informed versus a default reference genome. Paired t-test to test for significant difference in total reads mapped for the whole transcriptome, chromosome 8, and chromosome X. Nonparametric Wilcox single rank sum test was used to test for significant difference in total reads mapped on the Y chromosome for male samples in each tissue separately. Red * indicate a significant, p-value <0.05, difference in average mapped reads, NS is no significant differences. [file 13293_2020_312_MOESM7_ESM.pdf]

# STAR mapped reads

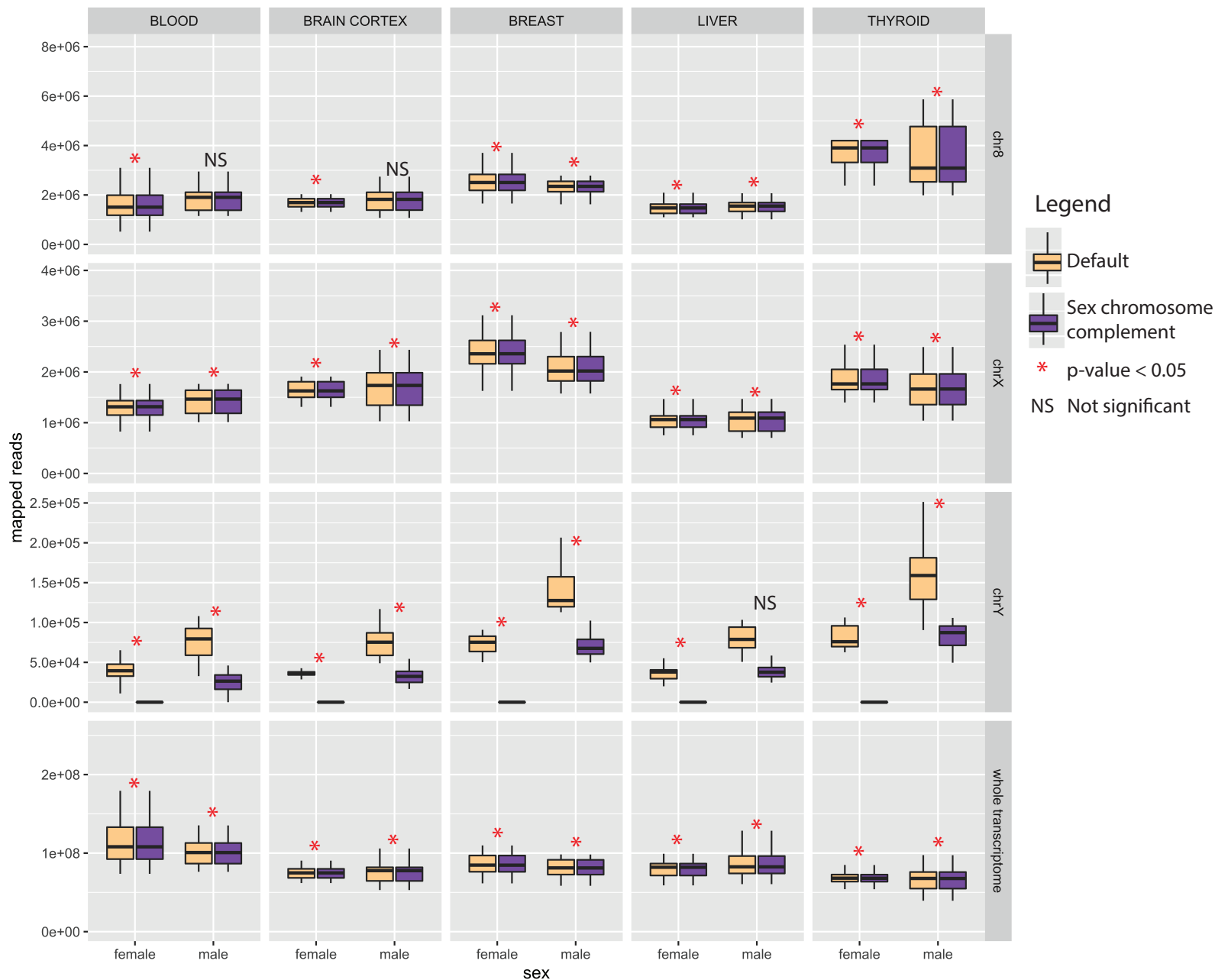

Supplement: Supplementary file 8 — Additional file 8: STAR mapped reads bar plot. Mean difference in expression for average total reads mapped for each tissue and each sex when aligned to a sex chromosome informed versus a default reference genome. Paired t-test to test for significant difference in total reads mapped for the whole transcriptome, chromosome 8, and chromosome X. Nonparametric Wilcox single rank sum test was used to test for significant difference in total reads mapped on the Y chromosome for male samples in each tissue separately. Red * indicate a significant, p-value <0.05, difference in average mapped reads, NS is no significant differences. [file 13293_2020_312_MOESM8_ESM.pdf]

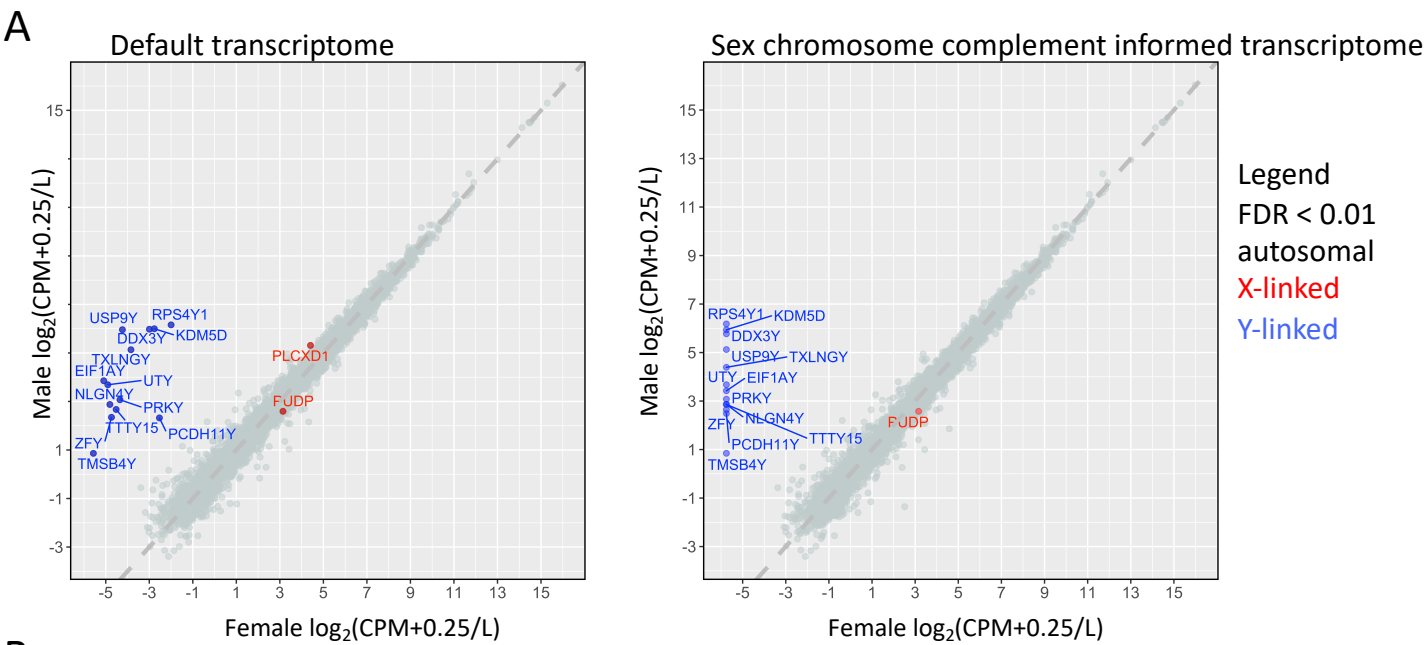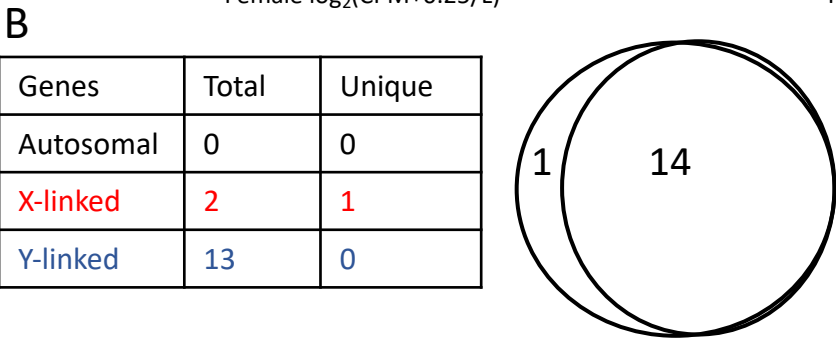

| Genes     | Total | Unique |
|-----------|-------|--------|
| Autosomal | 0     | 0      |
| X-linked  | 1     | 0      |
| Y-linked  | 13    | 0      |

Supplement: Supplementary file 18 — Additional file 18: Sex chromosome complement informed transcriptome reference eliminates Y-linked expression in female XX samples. A) Sex differences in gene expression, log2(CPM + 0.25/L), between the sixteen samples from genetic males and females are shown when aligning all samples to the default Ensembl reference transcriptome (left) and a reference transcriptome informed on the sex chromosome complement (right) for brain cortex. Each point represents a gene. Genes that are differentially expressed, adjusted p-value <0.01 are indicated in black for autosomal genes, blue for Y-linked genes, and red for X-linked genes. B) We show overlap between genes that are called as differentially expressed when all samples are pseudo-aligned to the default transcriptome, and genes that are called as differentially expressed when pseudo-aligned to a sex chromosome complement informed transcriptome reference. When samples were aligned to a reference transcriptome informed on the sex chromosome complement, 14 genes were called as differentially expressed between the sexes. PLCXD1 was uniquely called as differentially expressed when aligned to a default reference genome. Ensembl sex chromosome complement informed transcriptome reference eliminates Y-linked expression in female XX samples. A) Sex differences in gene expression, log2(CPM + 0.25/L), between the sixteen samples from genetic males and females are shown when aligning all samples to the default Ensembl reference transcriptome (left) and a reference transcriptome informed on the sex chromosome complement (right) for brain cortex. Each point represents a gene. Genes that are differentially expressed, adjusted p-value <0.01 are indicated in black for autosomal genes, blue for Y-linked genes, and red for X-linked genes. B) We show overlap between genes that are called as differentially expressed when all samples are pseudo-aligned to the default transcriptome, and genes that are called as differentially expressed when pseud [file 13293_2020_312_MOESM18_ESM.pdf]

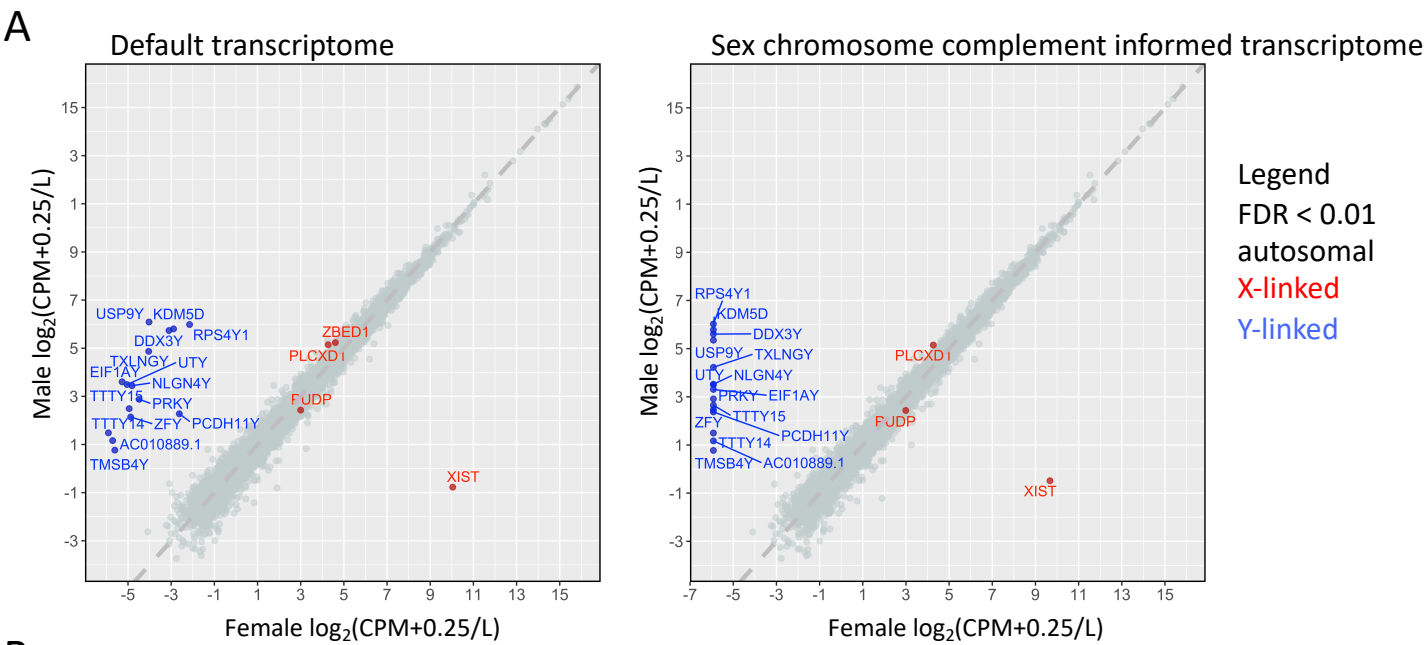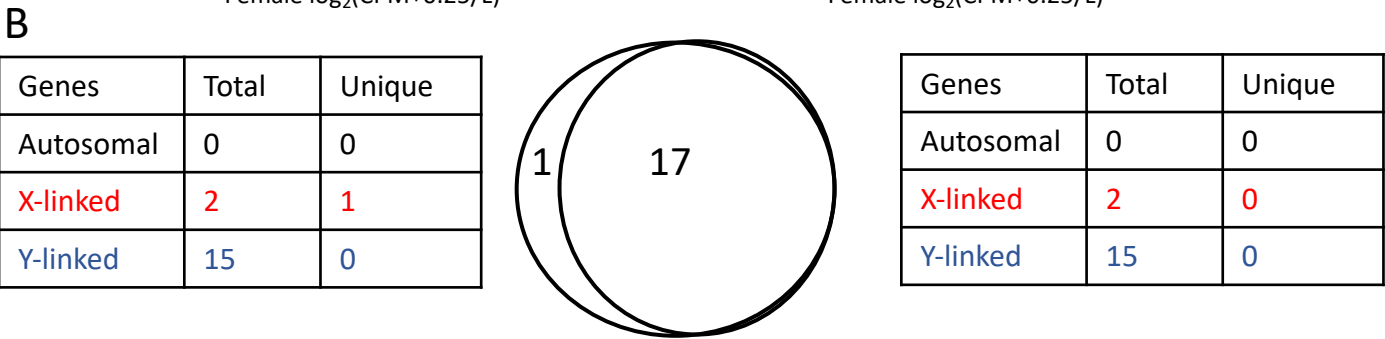

Supplement: Supplementary file 19 — Additional file 19: Gencode sex chromosome complement informed transcriptome reference eliminates Y-linked expression in female XX samples. A) Sex differences in gene expression, log2(CPM + 0.25/L), between the sixteen samples from genetic males and females are shown when aligning all samples to the default gencode reference transcriptome (left) and a reference transcriptome informed on the sex chromosome complement (right) for brain cortex. Each point represents a gene. Genes that are differentially expressed, adjusted p-value <0.01 are indicated in black for autosomal genes, blue for Y-linked genes, and red for X-linked genes. B) We show overlap between genes that are called as differentially expressed when all samples are pseudo-aligned to the default transcriptome, and genes that are called as differentially expressed when pseudo-aligned to a sex chromosome complement informed transcriptome reference. When samples were aligned to a reference transcriptome informed on the sex chromosome complement, 17 genes were called as differentially expressed between the sexes. ZBED1 was uniquely called as differentially expressed when aligned to a default reference genome. [file 13293_2020_312_MOESM19_ESM.pdf]
