# Supplementary material for: Reference genome and transcriptome informed by the sex chromosome complement of the sample increase ability to detect sex differences in gene expression from RNA-Seq data
Source: Biol Sex Differ. 2020 Jul 21;11:42. doi: 10.1186/s13293-020-00312-9 (PMC7374973; doi:10.1186/s13293-020-00312-9)
Supplement: Supplementary file 10 — Additional file 10: X chromosome expression differences between default and sex chromosome complement informed alignment. X chromosome gene expression differences between default and sex chromosome complement informed alignment. Increase in expression when aligned to a sex chromosome complement informed reference genome is a log2 fold change (FC) > 0. A decrease in expression when aligned to a sex chromosome complement informed reference genome is log2 FC < 0. Female XX samples are indicated by red and pink circles for PAR1, XTR, PAR2 genes, and for all other X chromosome genes respectively. Blue and light blue squares represent male XY samples. Blue squares indicate which gene points are in PAR1, XTR, and PAR2, and light blue squares are for genes outside of those regions. Differences in X chromosome expression between reference genomes default and sex chromosome complement for male XY and female XX samples aligned using HISAT for the whole X chromosome and the first 5 Mb are shown for the whole blood (A and B, respectively), brain cortex (E and F, respectively), breast (I and J, respectively), liver (M and N, respectively), and thyroid (Q and R, respectively). Differences in X chromosome expression between reference genomes for male XY and female XX samples aligned using STAR for the whole X chromosome and the first 5 Mb are shown for the whole blood (C and D, respectively), brain cortex (G and H, respectively), breast (K and L, respectively), liver (O and P, respectively), and thyroid (S and T, respectively). [file 13293_2020_312_MOESM10_ESM.pdf]

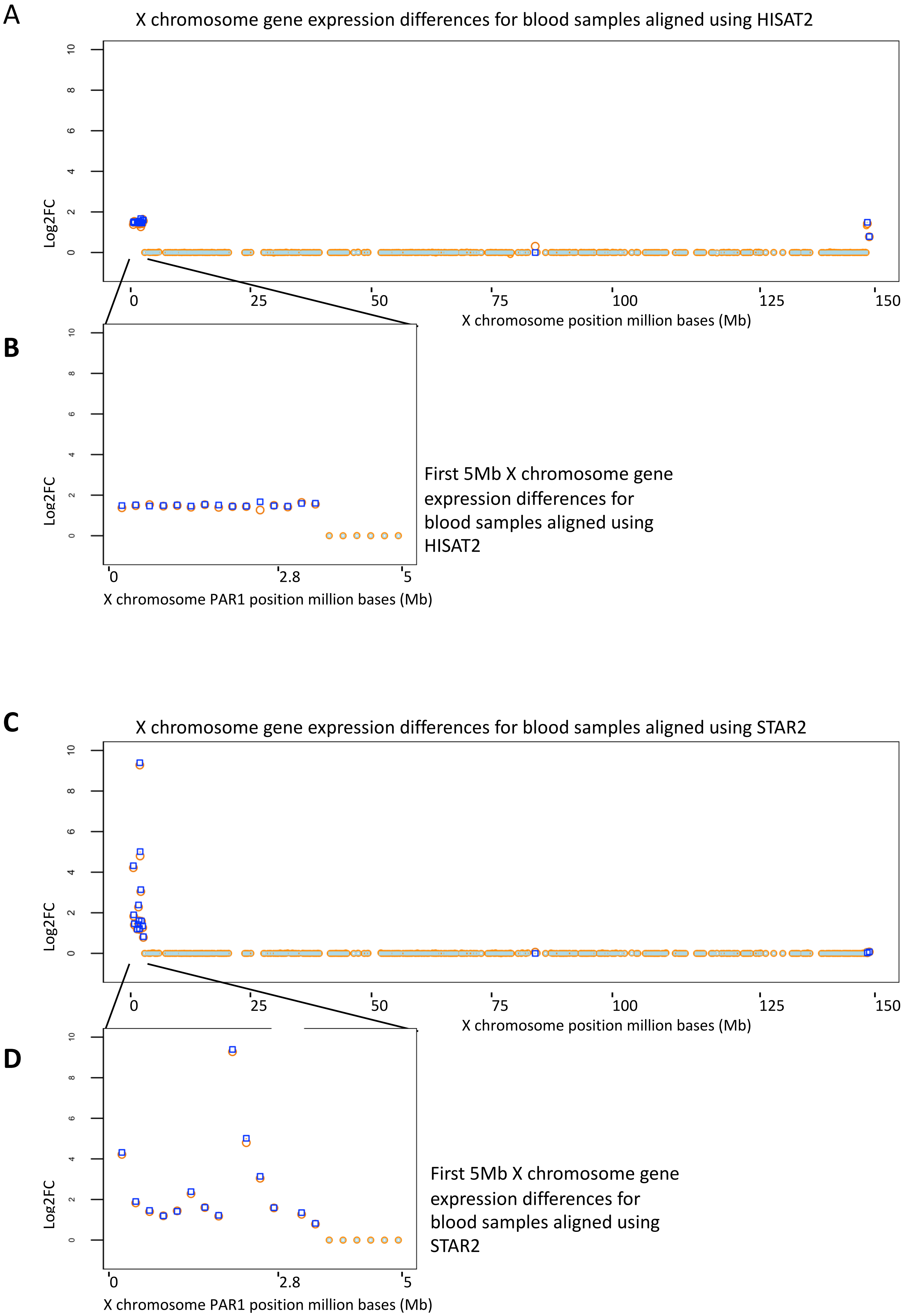

A
X chromosome gene expression differences for blood samples aligned using HISAT2
Log2FC
0
2
4
6
8
10
0
25
50
75
100
125
150
X chromosome position million bases (Mb)
B
Log2FC
0
2.8
5
X chromosome PAR1 position million bases (Mb)
First 5Mb X chromosome gene expression differences for blood samples aligned using HISAT2
C
X chromosome gene expression differences for blood samples aligned using STAR2
Log2FC
X chromosome position million bases (Mb)
D
Log2FC
X chromosome PAR1 position million bases (Mb)
First 5Mb X chromosome gene expression differences for blood samples aligned using STAR2

E

F

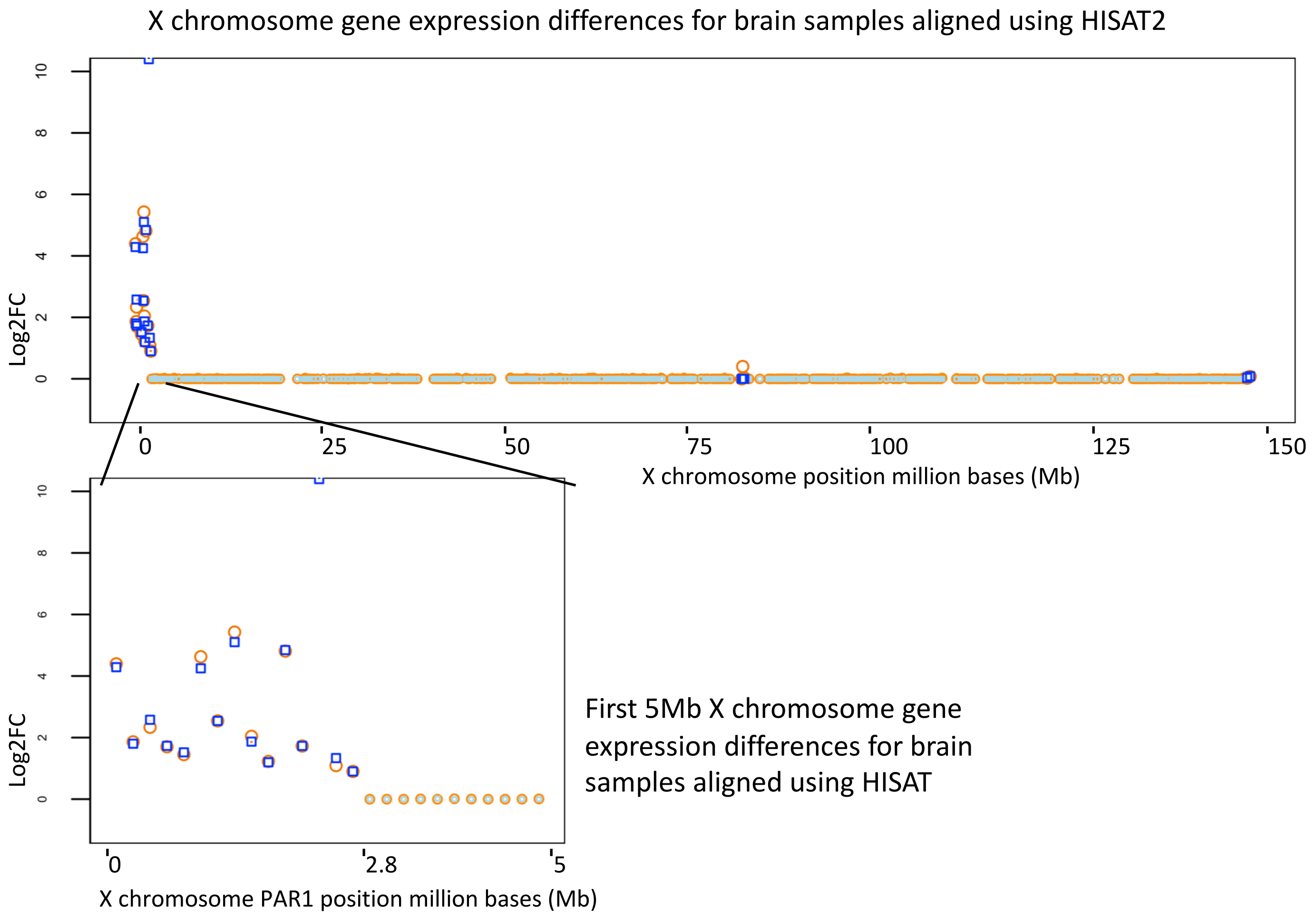

X chromosome gene expression differences for brain samples aligned using HISAT2
Log2FC
0
2
4
6
8
10
0
25
50
75
100
125
150
X chromosome position million bases (Mb)
Log2FC
0
2
4
6
8
10
0
2.8
5
X chromosome PAR1 position million bases (Mb)
First 5Mb X chromosome gene expression differences for brain samples aligned using HISAT

G

H

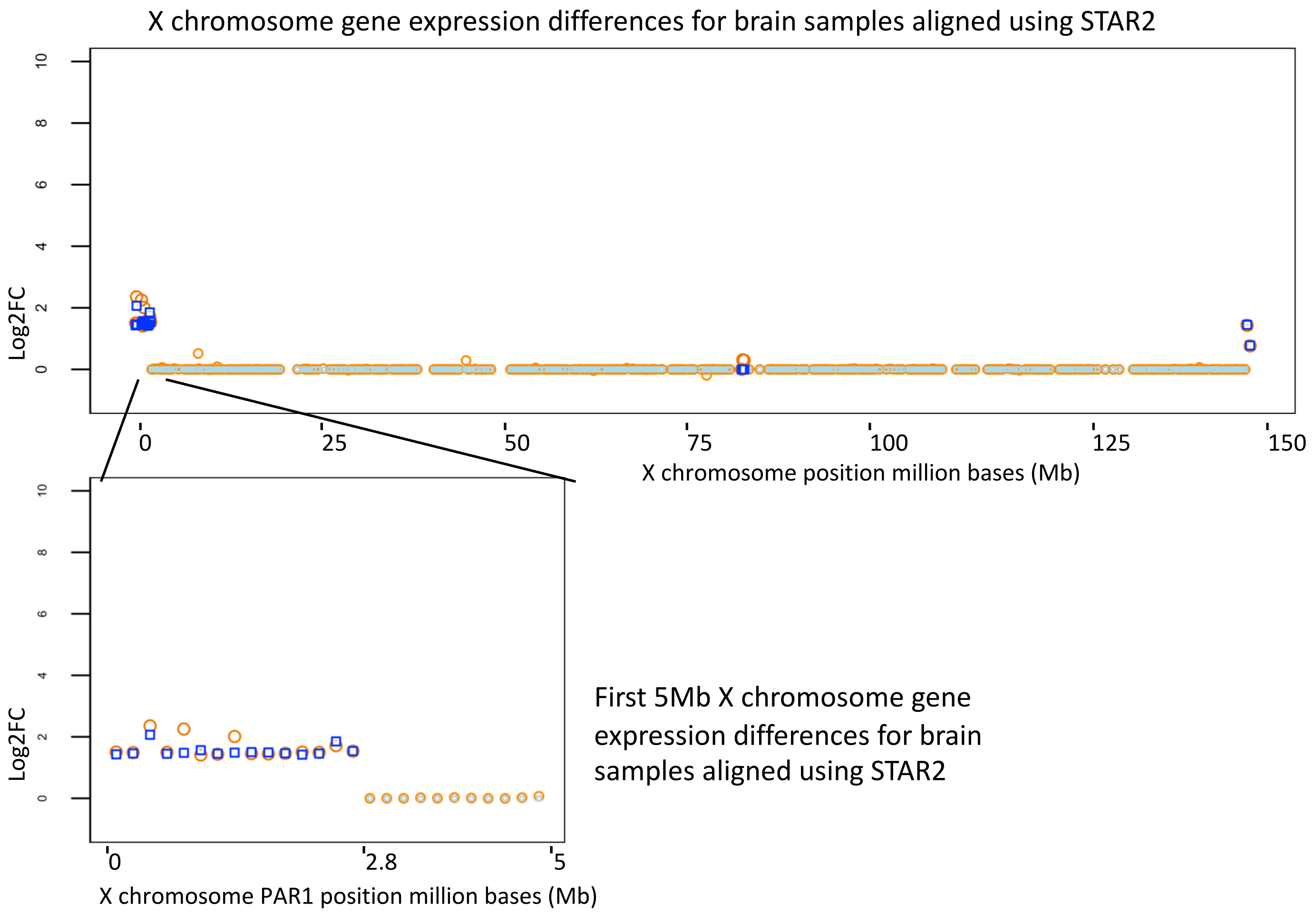

X chromosome gene expression differences for brain samples aligned using STAR2
Log2FC
0
2
4
6
8
10
0
25
50
75
100
125
150
X chromosome position million bases (Mb)
Log2FC
0
2
4
6
8
10
0
2.8
5
X chromosome PAR1 position million bases (Mb)
First 5Mb X chromosome gene expression differences for brain samples aligned using STAR2

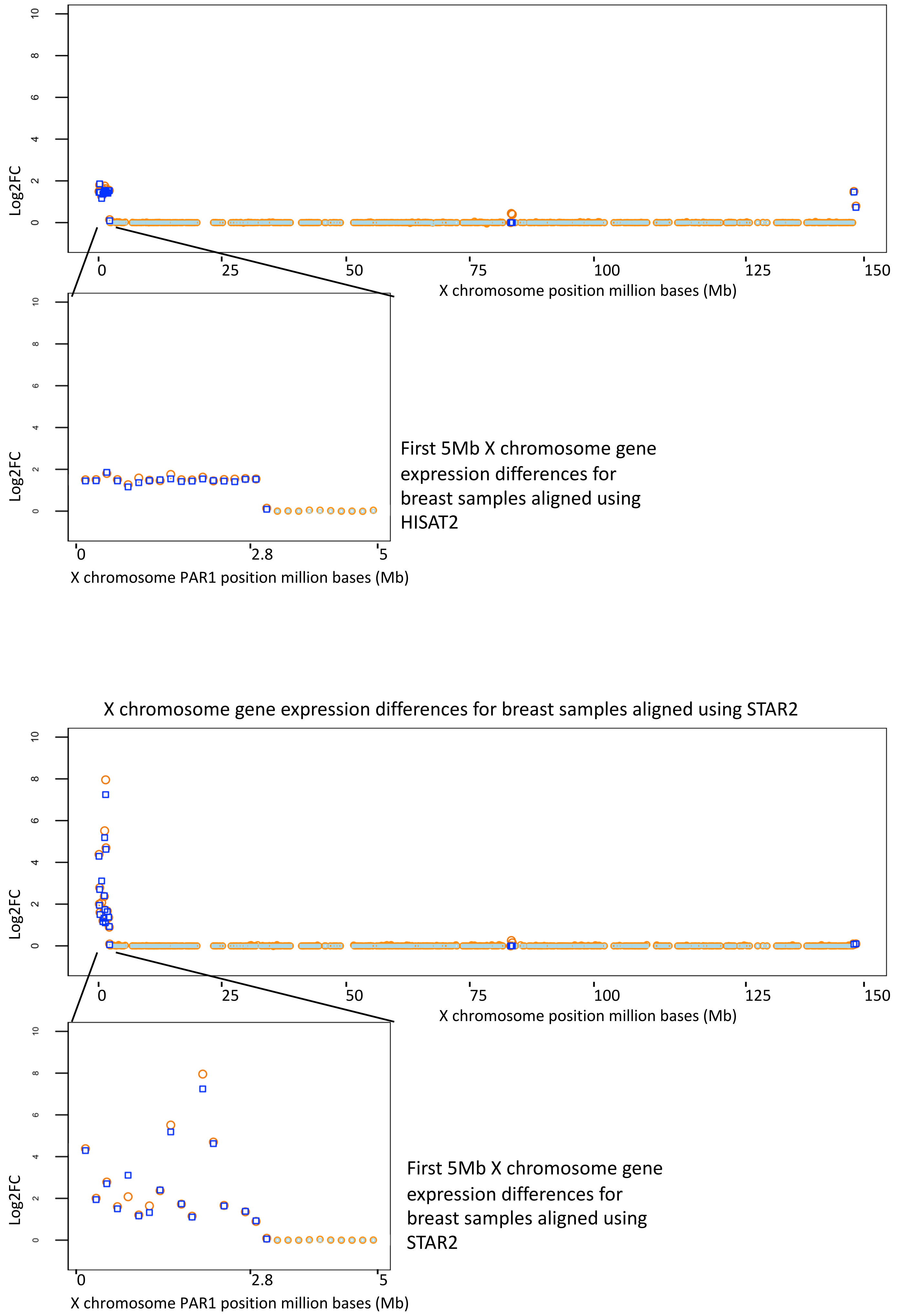
I
X chromosome gene expression differences for breast samples aligned using HISAT2
Log2FC
X chromosome position million bases (Mb)
0
25
50
75
100
125
150
J
Log2FC
X chromosome PAR1 position million bases (Mb)
0
2.8
5
First 5Mb X chromosome gene expression differences for breast samples aligned using HISAT2
K
X chromosome gene expression differences for breast samples aligned using STAR2
Log2FC
X chromosome position million bases (Mb)
0
25
50
75
100
125
150
L
Log2FC
X chromosome PAR1 position million bases (Mb)
0
2.8
5
First 5Mb X chromosome gene expression differences for breast samples aligned using STAR2

M

N

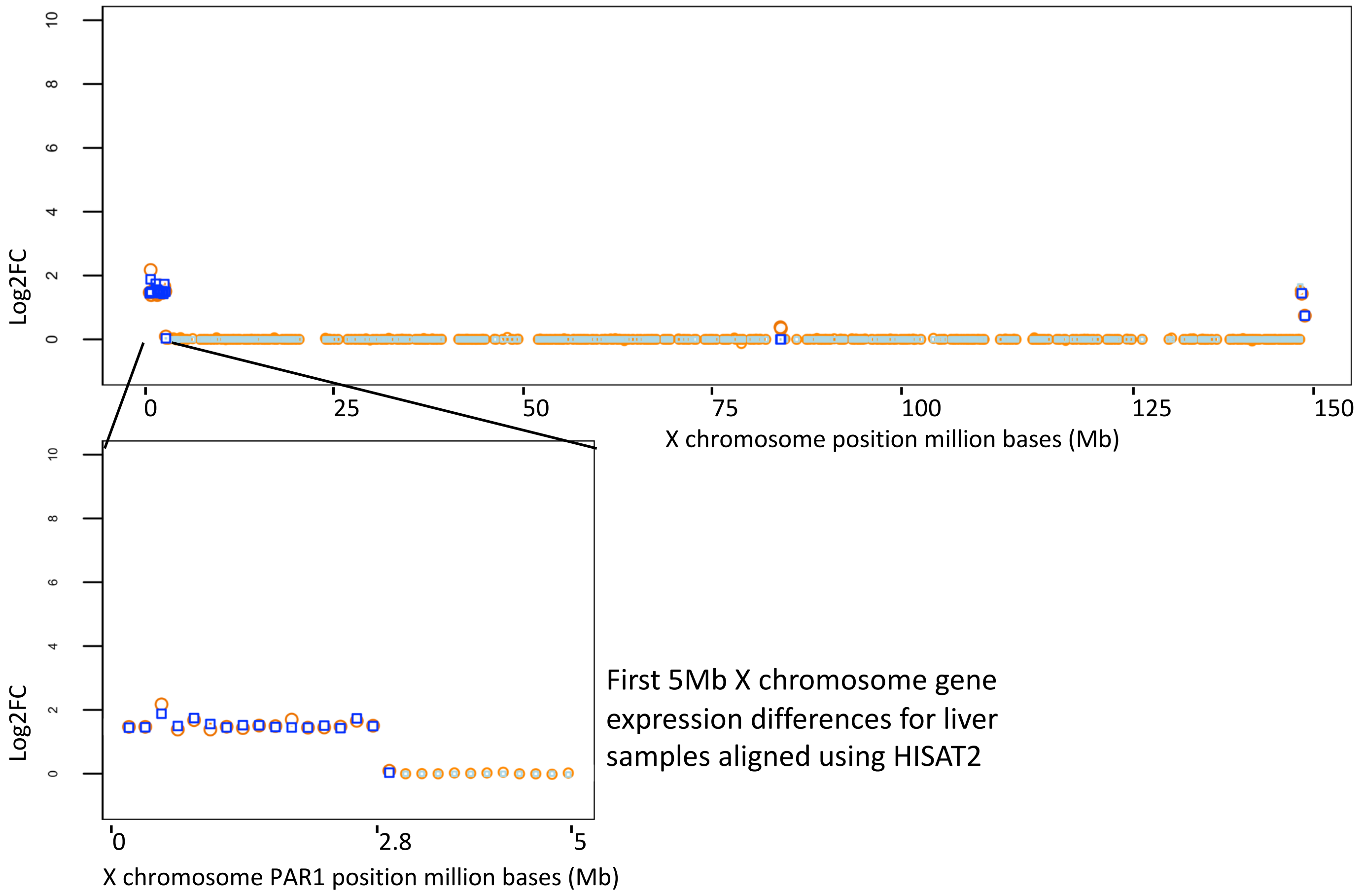
X chromosome gene expression differences for liver samples aligned using HISAT2
Log2FC
X chromosome position million bases (Mb)
First 5Mb X chromosome gene expression differences for liver samples aligned using HISAT2
X chromosome PAR1 position million bases (Mb)

O

P

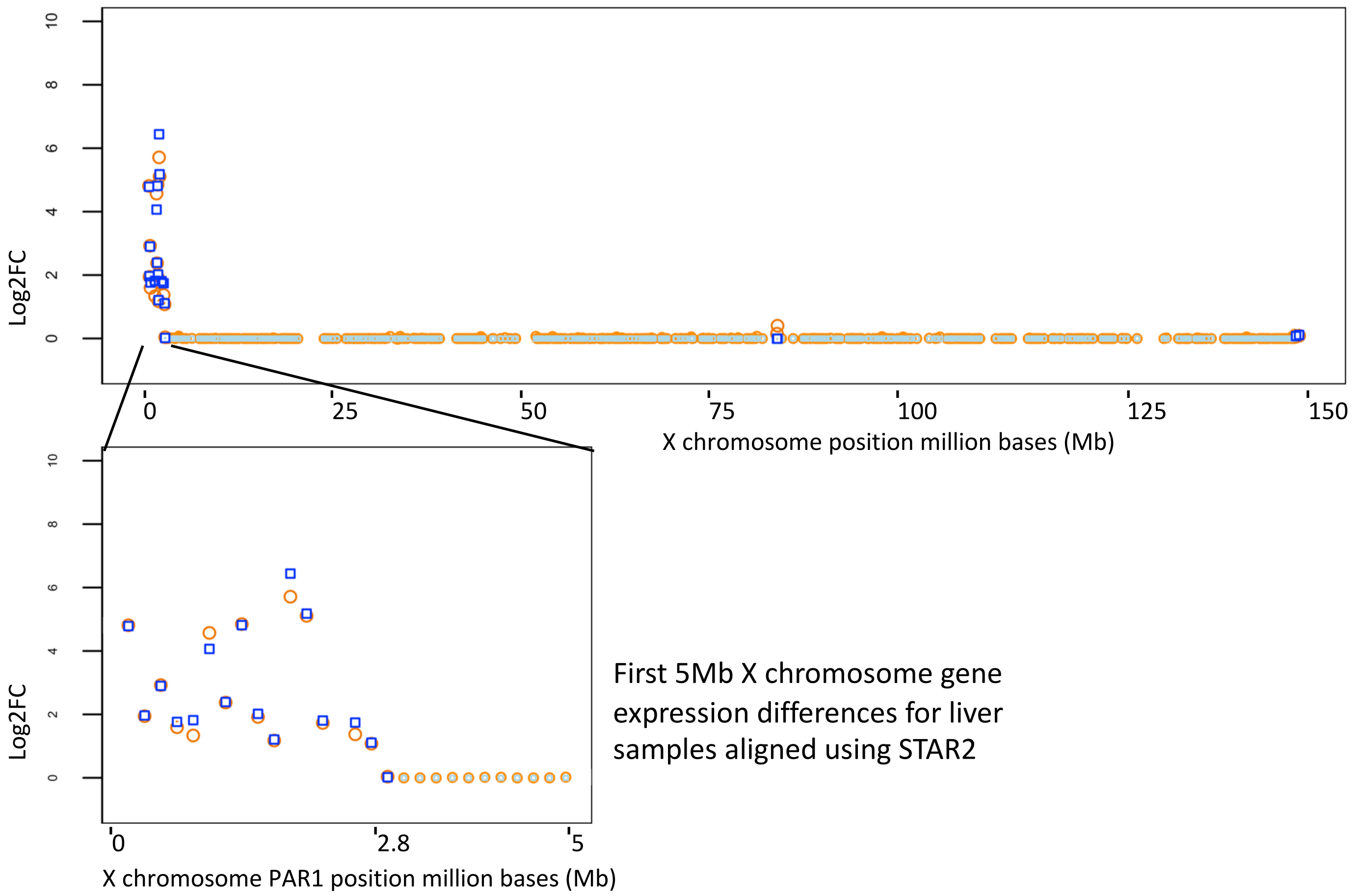
X chromosome gene expression differences for liver samples aligned using STAR2
Log2FC
X chromosome position million bases (Mb)
First 5Mb X chromosome gene expression differences for liver samples aligned using STAR2
X chromosome PAR1 position million bases (Mb)

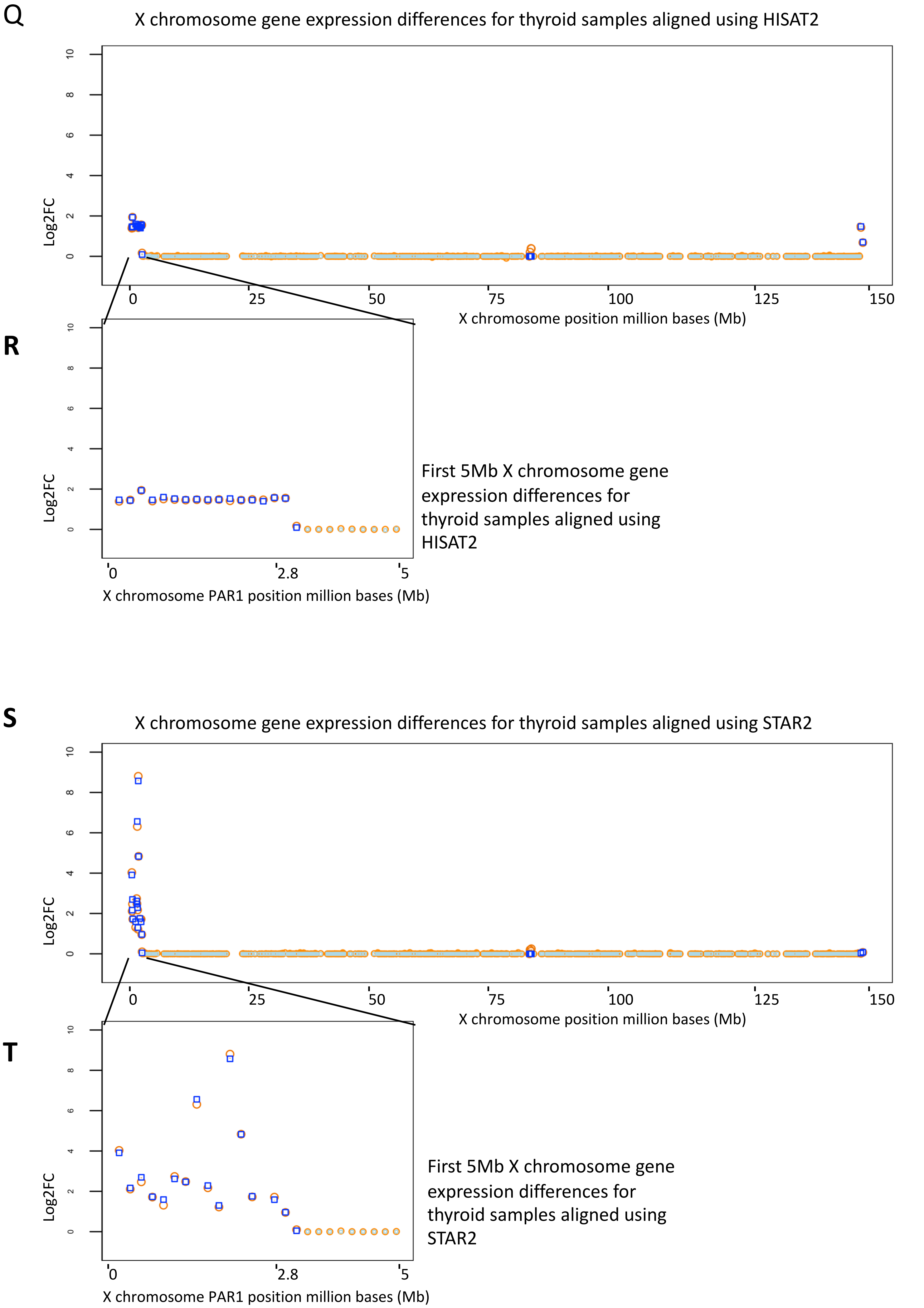
Q
X chromosome gene expression differences for thyroid samples aligned using HISAT2
Log2FC
0 2 4 6 8 10
0 25 50 75 100 125 150
X chromosome position million bases (Mb)
R
Log2FC
0 2 4 6 8 10
0 2.8 5
X chromosome PAR1 position million bases (Mb)
First 5Mb X chromosome gene expression differences for thyroid samples aligned using HISAT2
S
X chromosome gene expression differences for thyroid samples aligned using STAR2
Log2FC
0 2 4 6 8 10
0 25 50 75 100 125 150
X chromosome position million bases (Mb)
T
Log2FC
0 2 4 6 8 10
0 2.8 5
X chromosome PAR1 position million bases (Mb)
First 5Mb X chromosome gene expression differences for thyroid samples aligned using STAR2
