# Supplementary material for: Reference genome and transcriptome informed by the sex chromosome complement of the sample increase ability to detect sex differences in gene expression from RNA-Seq data
Source: Biol Sex Differ. 2020 Jul 21;11:42. doi: 10.1186/s13293-020-00312-9 (PMC7374973; doi:10.1186/s13293-020-00312-9)
Supplement: Supplementary file 12 — Additional file 12: Whole genome gene expression values per sample, aligner and reference genome used for alignment. CPM values for male XY and female XX whole blood, brain cortex, breast, liver and thyroid samples when aligned to a default and sex chromosome complement informed reference genome for the whole genome (1-22, mtDNA, X, Y and non-chromosomal). [file 13293_2020_312_MOESM12_ESM.docx]

## **Additional File 14**

Additional File 14 is a series of HISAT and STAR output quantification files. Files will be available for download via Dryad upon publication.
