# Supplementary material for: Reference genome and transcriptome informed by the sex chromosome complement of the sample increase ability to detect sex differences in gene expression from RNA-Seq data
Source: Biol Sex Differ. 2020 Jul 21;11:42. doi: 10.1186/s13293-020-00312-9 (PMC7374973; doi:10.1186/s13293-020-00312-9)

A Gene expression differences between male XY and female XX blood samples aligned using HISAT

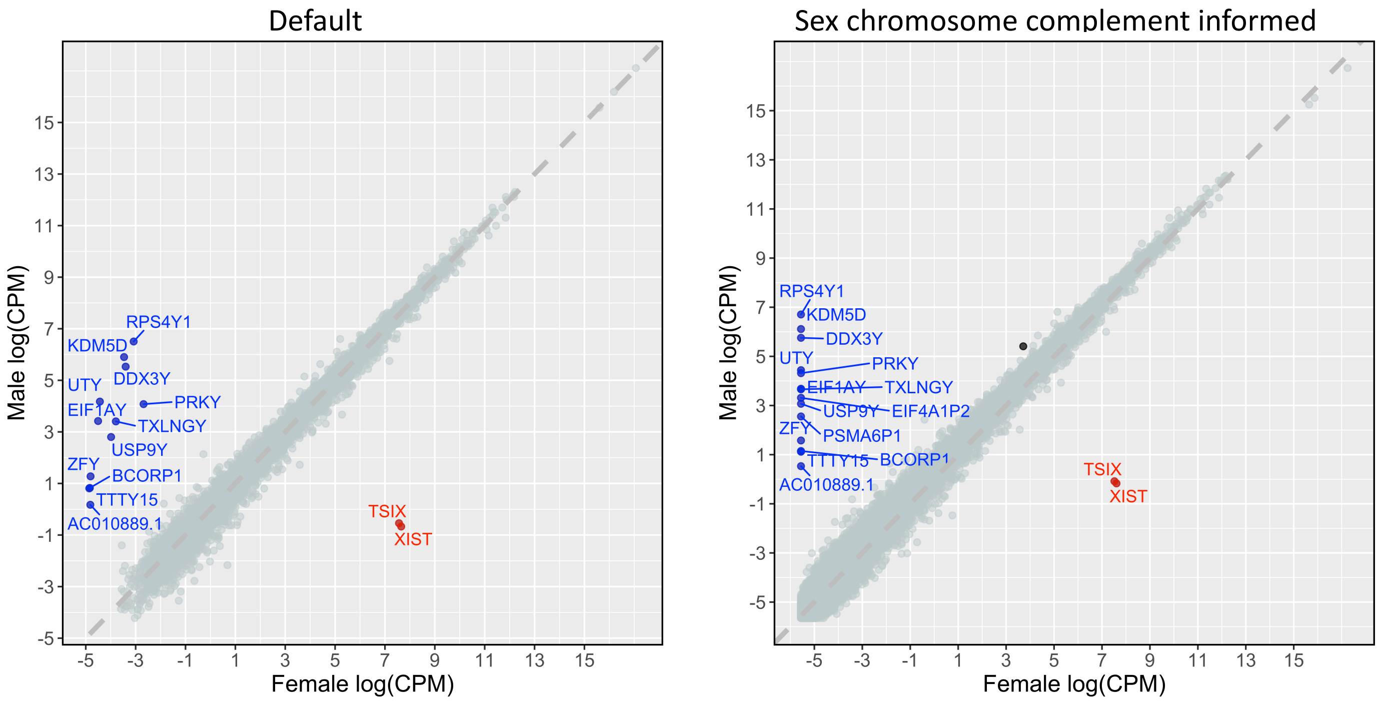

B Gene expression differences between male XY and female XX blood samples aligned using STAR

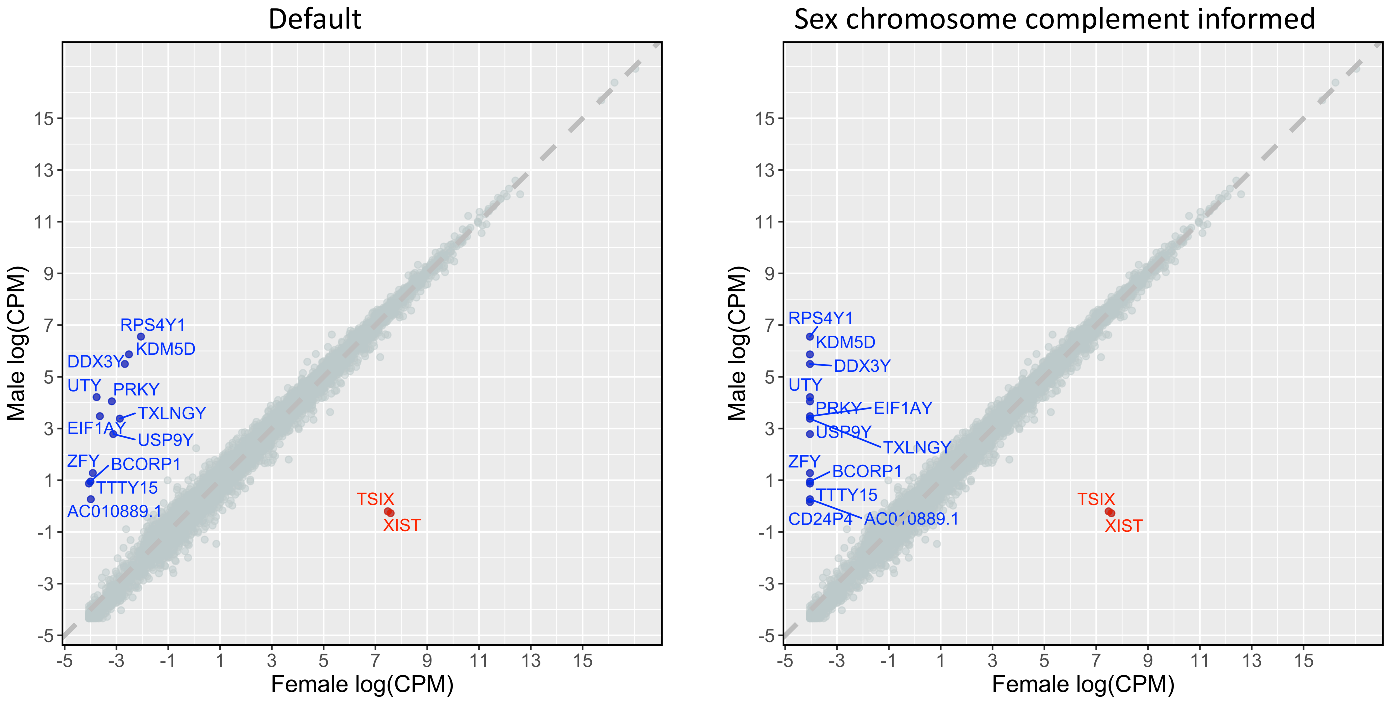

C Gene expression differences between male XY and female XX brain samples aligned using HISAT

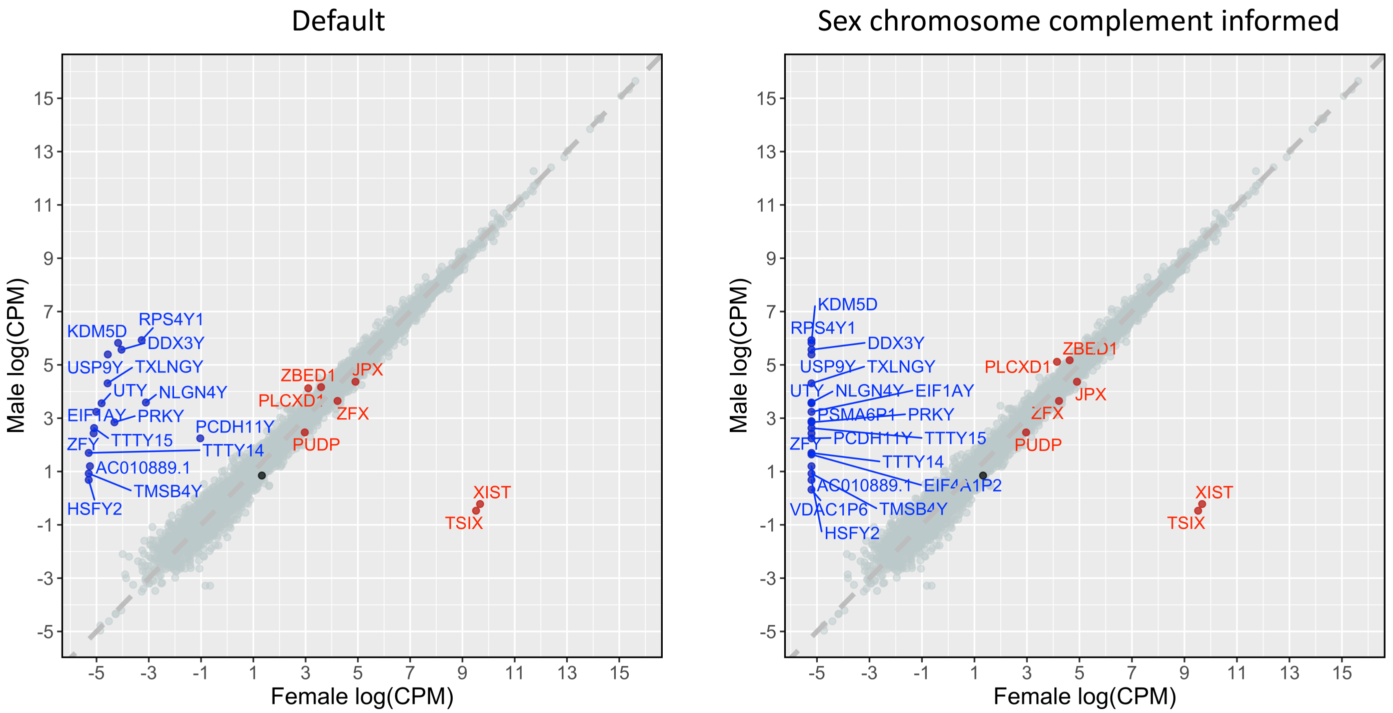

D Gene expression differences between male XY and female XX brain samples aligned using STAR

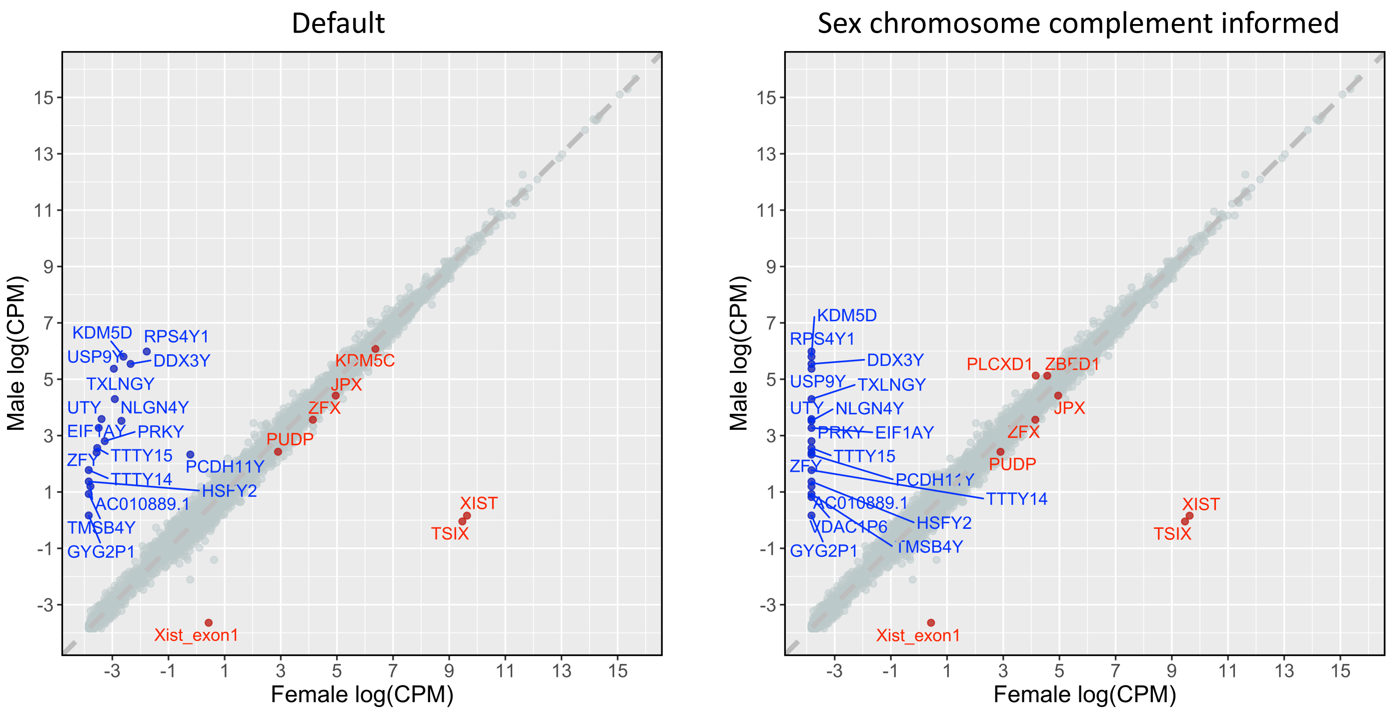

E Gene expression differences between male XY and female XX breast samples aligned using HISAT

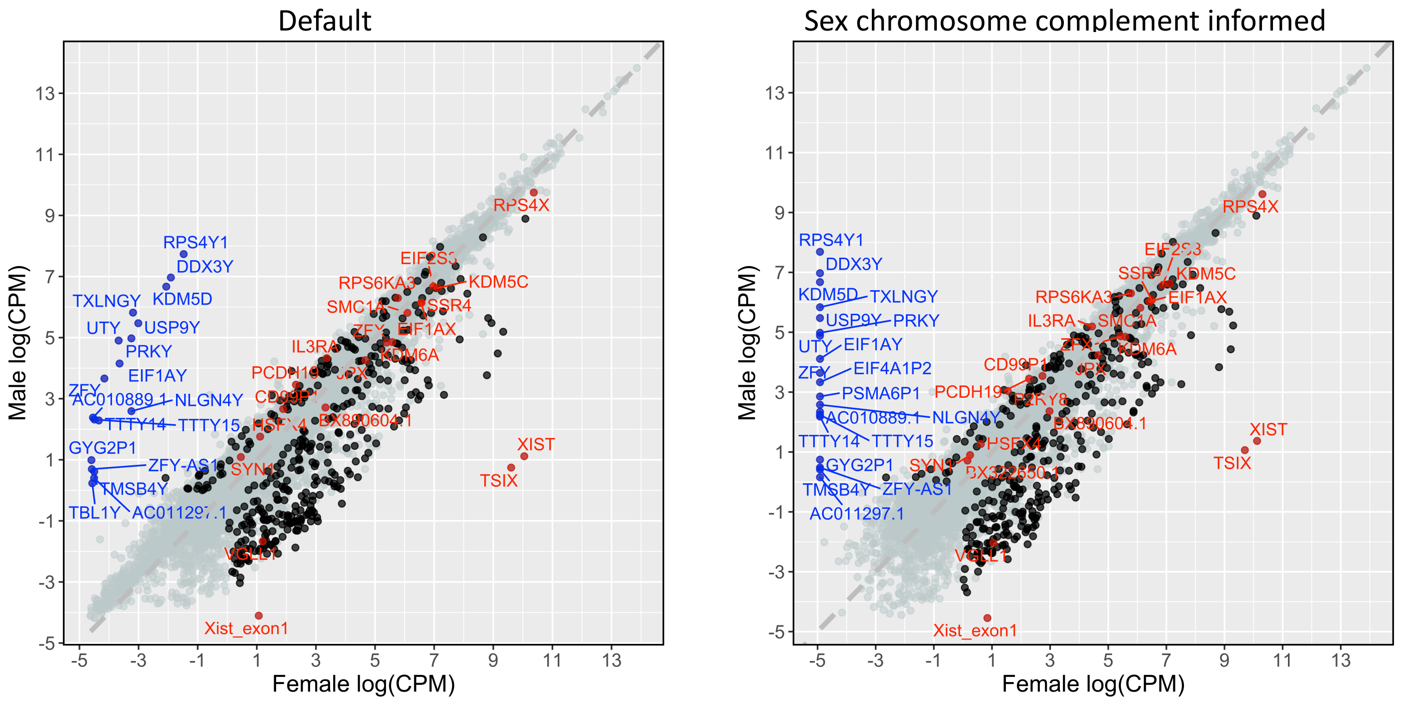

F Gene expression differences between male XY and female XX breast samples aligned using STAR

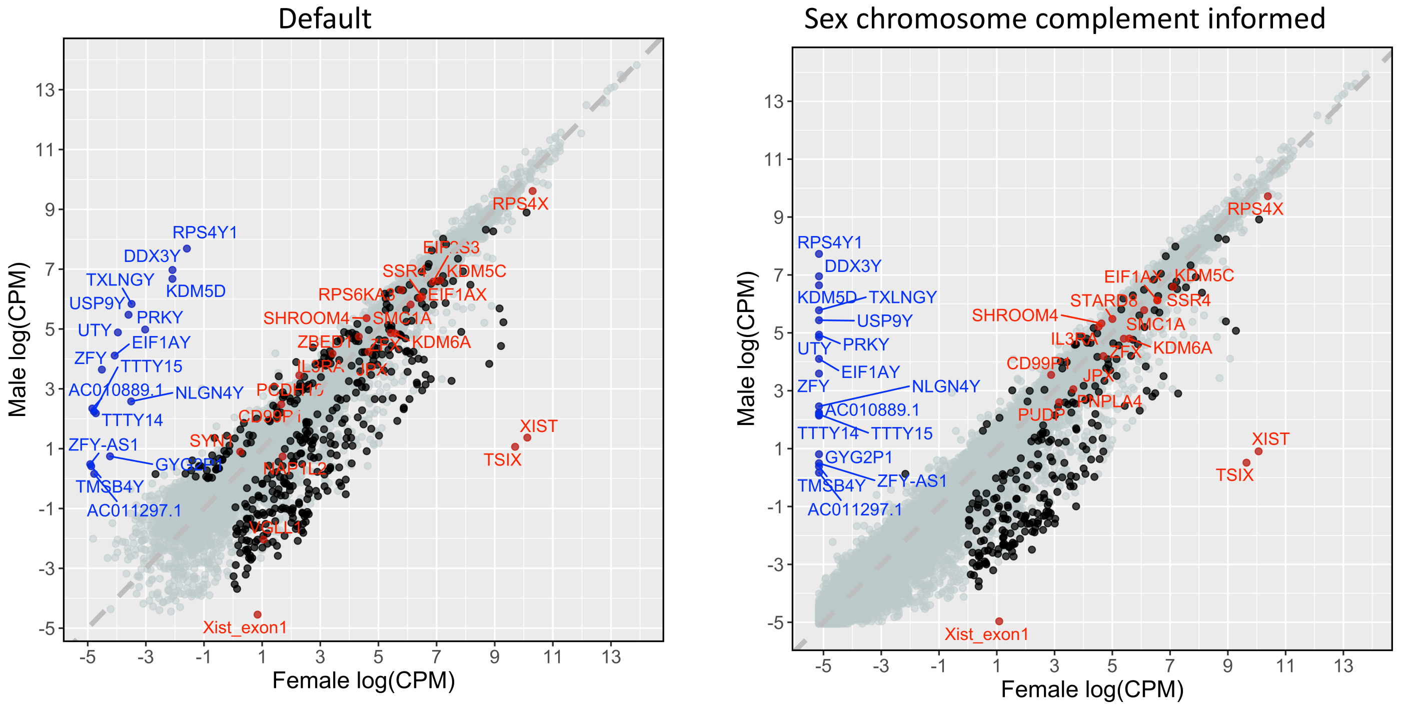

G Gene expression differences between male XY and female XX liver samples aligned using HISAT

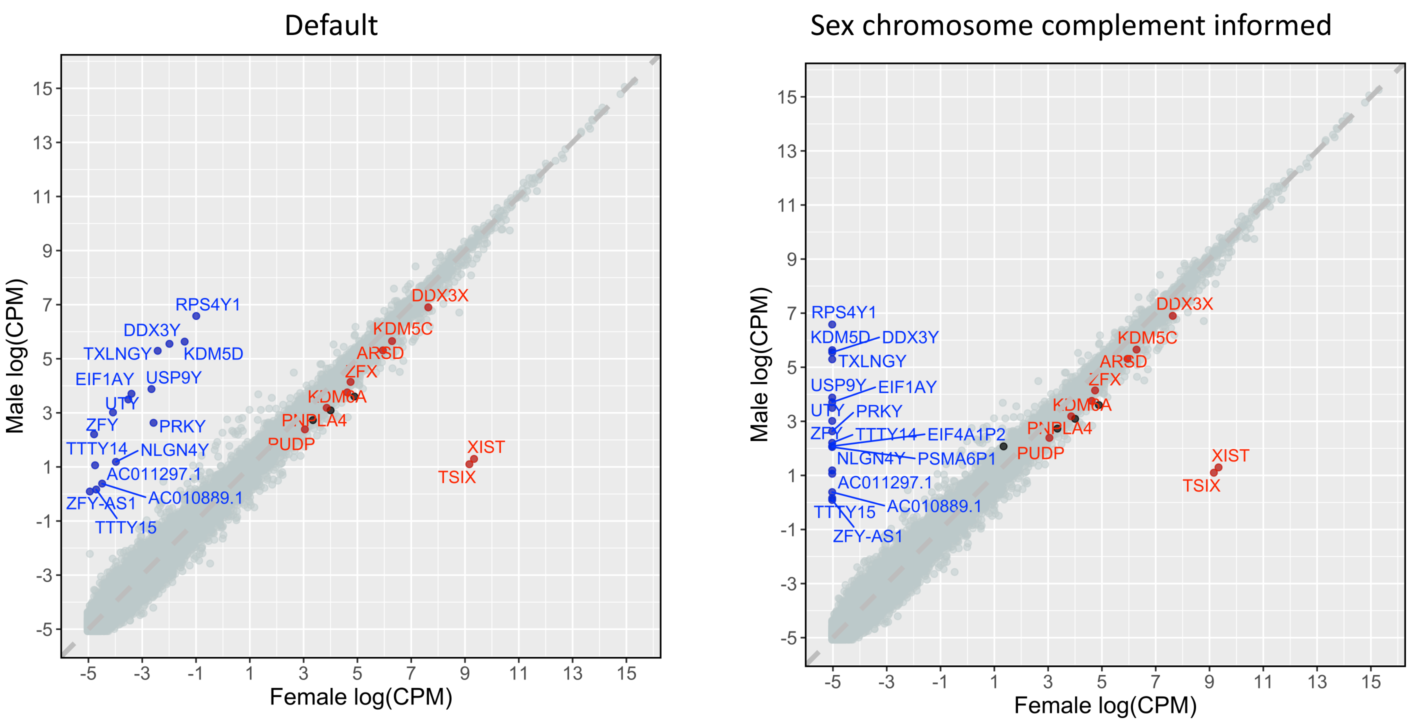

H Gene expression differences between male XY and female XX liver samples aligned using STAR

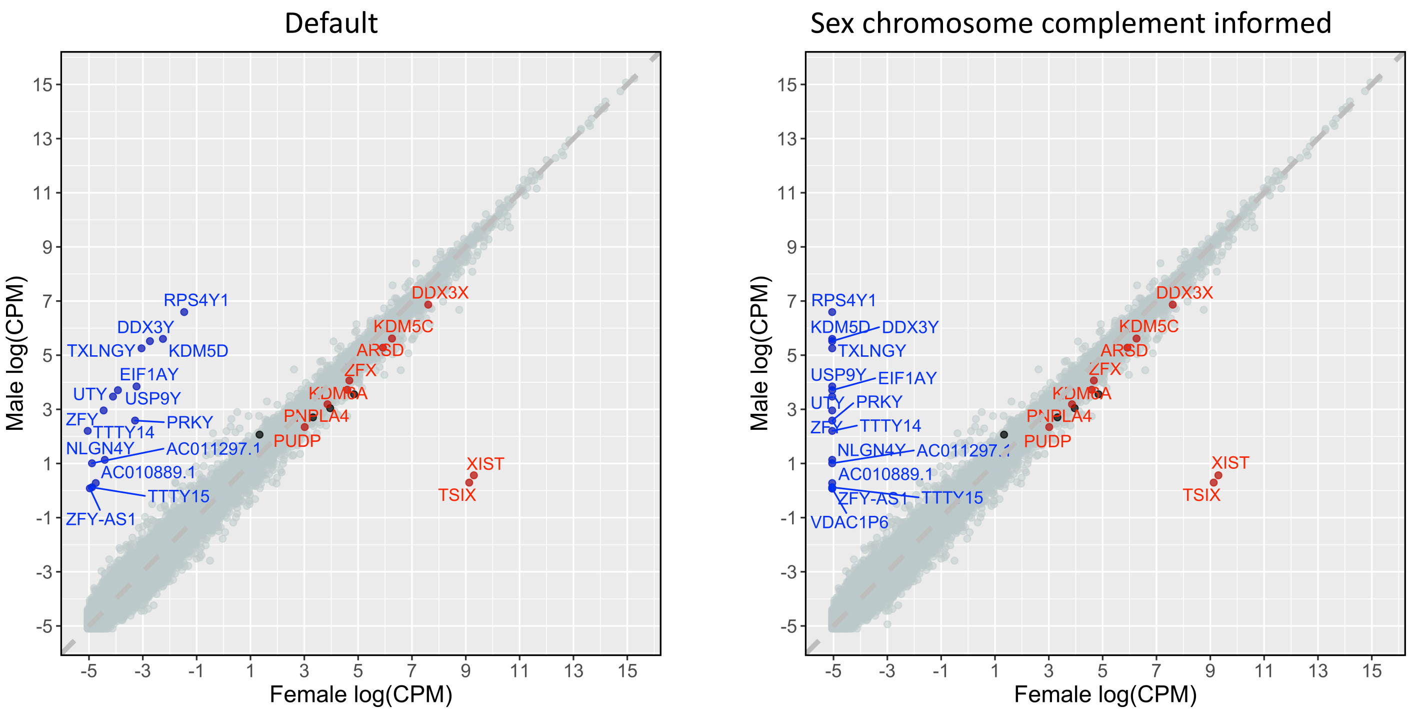

I Gene expression differences between male XY and female XX thyroid samples aligned using HISAT

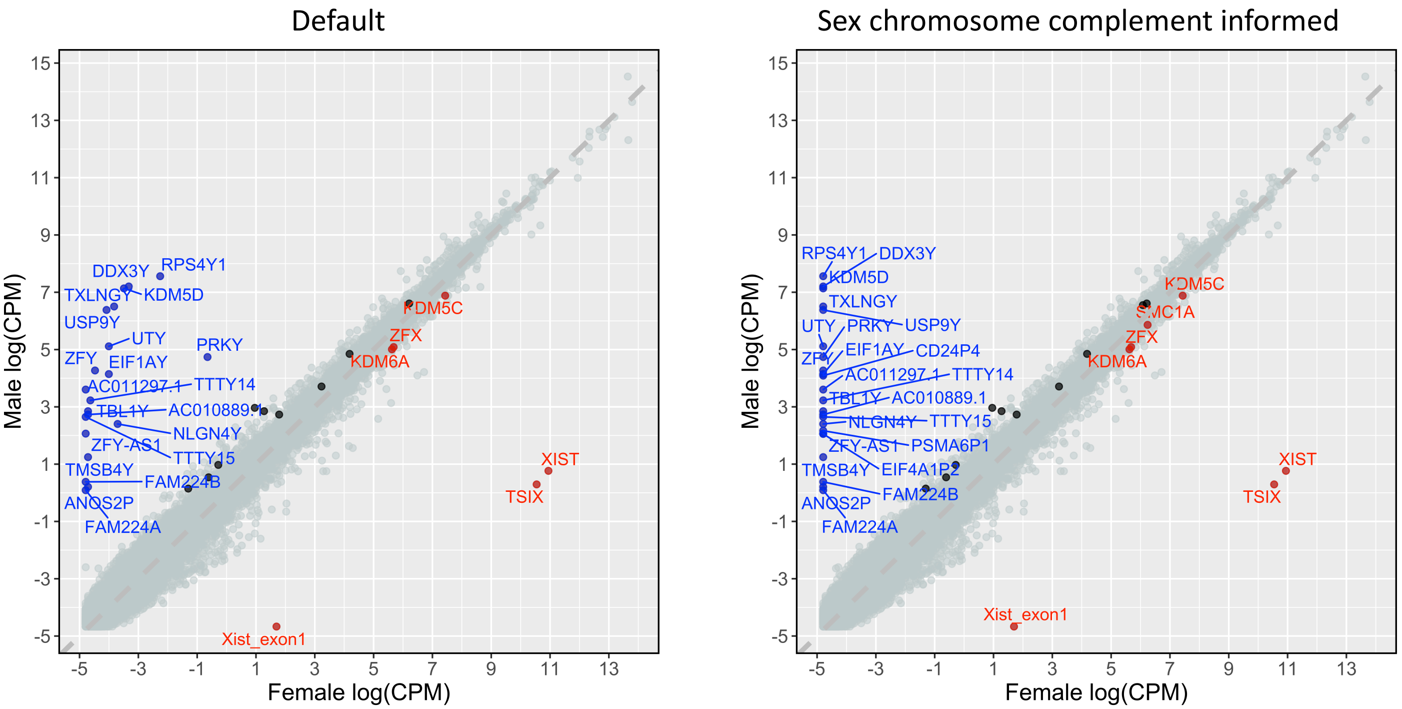

J Gene expression differences between male XY and female XX thyroid samples aligned using STAR

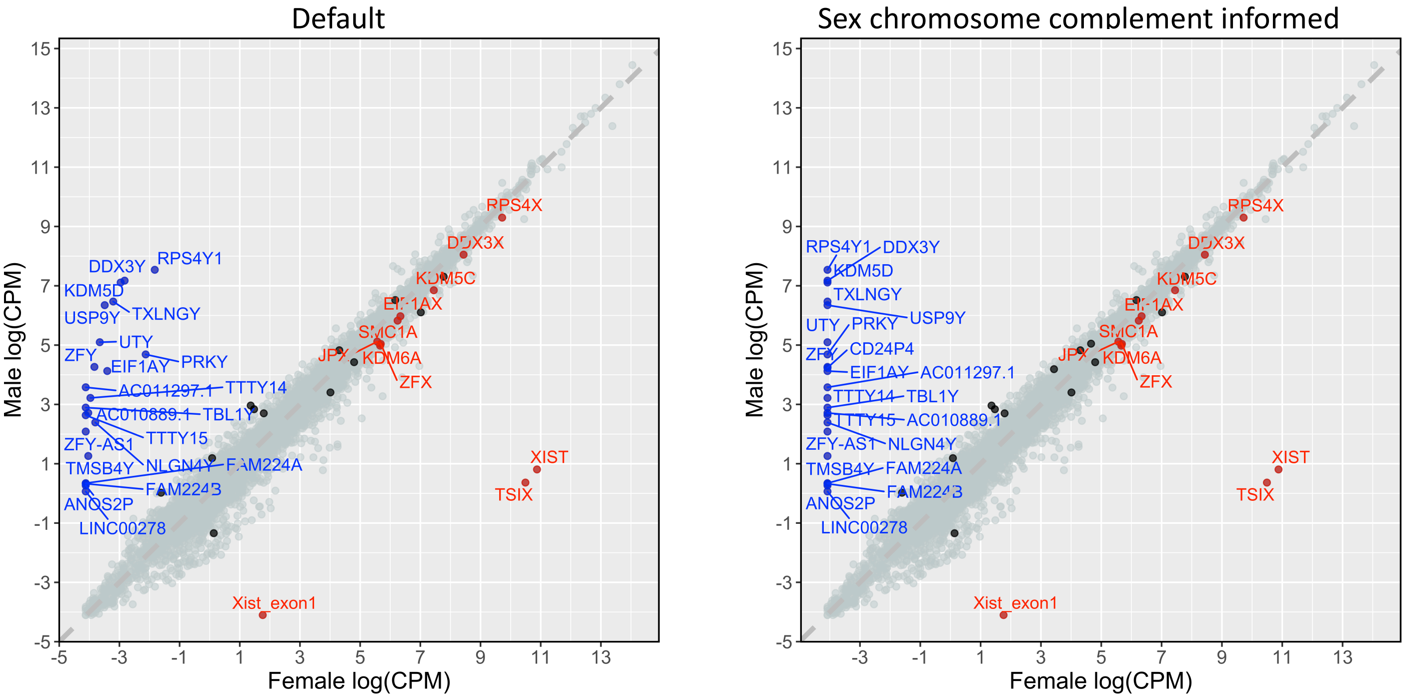

Supplement: Supplementary file 15 — Additional file 15: Gene expression differences between male XY and female XX samples. Sex differences in gene expression for whole blood, brain cortex, breast, liver, and thyroid samples for when samples were aligned to a default reference genome and to a reference genome informed on the sex chromosome complement. Showing sex differences in gene expression between reference genomes used for alignment and for when samples were aligned using HISAT and STAR. [file 13293_2020_312_MOESM15_ESM.pdf]
